# *Ex vivo-*generated lymphoid progenitors encompass both T cell and innate lymphoid cell fates

**DOI:** 10.3389/fimmu.2025.1617707

**Published:** 2025-07-23

**Authors:** Pierre Gaudeaux, Juliette Paillet, Monah Abou Alezz, Ranjita Devi Moirangthem, Sara Cascione, Marta Martin Corredera, Anne-Catherine Dolens, Katrien De Mulder, Imke Velghe, Bart Vandekerckhove, Marieke Lavaert, Noémie Robil, Aurélien Corneau, Hanem Sadek, Pauline Rault, Akshay Joshi, Pierre de la Grange, Frank J. T. Staal, Tom Taghon, Olivier Negre, Andrea Ditadi, Isabelle André, Tayebeh-Shabi Soheili

**Affiliations:** ^1^ Laboratory of Human Lymphohematopoieisis, Imagine Institute, INSERM UMR 1163, Université Paris Cité, Paris, France; ^2^ Smart Immune, Paris, France; ^3^ San Raffaele Telethon Institute for Gene Therapy, IRCCS San Raffaele Scientific Institute, Milan, Italy; ^4^ Cancer Research Institute Ghent (CRIG), Ghent University, Ghent, Belgium; ^5^ T Cell Team Taghon, Department of Diagnostic Sciences, Ghent University, Ghent, Belgium; ^6^ GenoSplice, Paris, France; ^7^ Centre de recherche Saint-Antoine, CISA, Sorbonne Université, Paris, France; ^8^ Cytometry Core Facility CyPS, Sorbonne Université, Paris, France; ^9^ Department of Pediatrics, Pediatric Stem Cell Transplantation Program, Willem-Alexander Children’s Hospital, Leiden, Netherlands; ^10^ Department of Immunology, Leiden University Medical Center, Leiden, Netherlands

**Keywords:** T cell progenitors, lymphoid cell development, scRNAseq, ex vivo differentiation, hematopoietic stem cell

## Abstract

**Introduction:**

We previously established a feeder-free cell therapy platform for the *ex vivo* generation of lymphoid-primed progenitors using immobilized Delta-like ligand 4 (DLL4). *In vivo* studies demonstrated that adoptive transfer of these progenitors accelerates T cell reconstitution following thymic engraftment.

**Method:**

To further explore the full therapeutic potential of this cell product, we performed a comprehensive molecular and phenotypic characterization using single cell RNA sequencing and mass cytometry analysis.

**Results:**

Our analysis revealed the presence of distinct cell subsets within the cellular product characterized mainly by commitment to lymphoid lineages. Using integrated transcriptomic analyses to compare these *ex vivo*-generated progenitors to *in vivo* human thymocytes, we revealed strong similarities with early stages of T cell development, underscoring the physiological relevance of our system. We also delineated two distinct developmental trajectories within the CD7^+^ progenitor population: a T cell-oriented path, marked by CD5 upregulation, and an innate lymphoid cell (ILC)-oriented branch, identified by CD161 expression and an ILC-like gene signature. Despite these lineage predispositions, both subsets demonstrated plasticity, retaining the ability to differentiate into both T cells and natural killer (NK) cells *in vitro*. Additionally, in our experimental setting, we observed that BCL11B, a transcription factor essential for T cell commitment, regulates negatively myeloid cell differentiation while preserving the potential for NK cell development.

**Conclusion:**

These findings underscore the versatility of DLL4-based lymphoid progenitors in generating either T cells or ILCs in response to environmental cues. This research paves the way for innovative cell therapy approaches to treat immune deficiencies and cancer- and age-related immune dysfunctions.

## Graphical Abstract

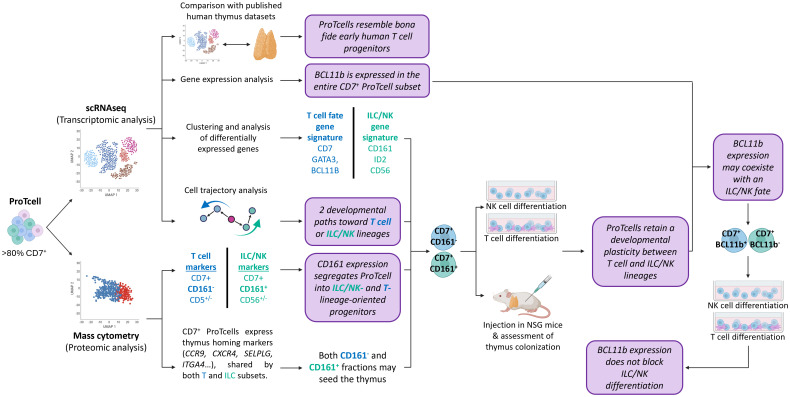

## Introduction

Immune response can be impaired by multiple factors, including specific genetic disorders or myeloablative chemotherapy regimens required for the treatment of hematologic malignancies. After allogeneic hematopoietic stem cell transplantation (HSCT), the standard of care for such pathologies, reconstituting an adaptive immune response is a time-consuming process (1, 2). Prolonged immunodeficiency following HSCT remains a significant challenge, increasing susceptibility to infectious complications and relapses (3–5).

There is a growing interest in the adoptive transfer of hematopoietic progenitors already committed to the T lymphoid lineage to accelerate T cell recovery after HSCT. T cells are derived from bone marrow progenitors that home to the thymus, where thymic epithelial cells provide essential signals for T cell differentiation and maturation. This thymic environment can be mimicked *in vitro* using murine stromal cell line engineered to express human Notch1 ligands, such as Delta-like ligand 1 (DLL1) or Delta-like ligand 4 (DLL4) (6, 7). Various studies have evaluated the *ex vivo* generation of T cell-committed progenitors for *in vivo* transplantation to enhance T cell reconstitution (8–12). It should be noted that feeder cell-free alternatives are important for clinical applications of lymphoid progenitors produced *ex vivo*; the use of murine stromal cells does not comply with good manufacturing practice (GMP) criteria and fails to meet standardization requirements.

In line with these efforts, we have developed a feeder cell-free therapy platform for *ex vivo* expansion and differentiation of human CD34^+^ hematopoietic stem and progenitor cells (HSPCs) into lymphoid-primed progenitors (13–15). This GMP-compliant process involves immobilization of the DLL4 protein combined with specific cytokines. Over a seven-day period, this approach produces a high yield of clinically applicable lymphoid progenitors referred to as ProTcells. The transplantation of these *ex vivo*-generated lymphoid progenitors into immunodeficient mice results in thymus colonization and the generation of mature polyclonal and functional T cells with an accelerated kinetic than is observed in conventional HSCT (13). Additionally, this platform accommodates CD34^+^ cells sourced from either cord blood (CB) or mobilized peripheral blood (mPB), highlighting its flexibility and utility (16). This platform is currently being evaluated in phase I/II clinical trials for treating malignant hematologic disorders (17).

Despite the strong evidence supporting the thymic homing and T cell potential of *ex vivo*-generated lymphoid progenitors, several questions remain unanswered. First, the comparability of ProTcells to bona fide natural thymus seeding progenitor cells is not fully understood. In addition, establishing a thorough characterization of the cell therapy product, including its heterogeneity, will be vital for elucidating its mechanism of action and deciphering clinical outcomes.

In this study, we employed single cell RNA sequencing (scRNAseq) and mass cytometry (CyTOF for cytometry by time of flight) to conduct an in-depth characterization of gene and protein expression profiles, as well as the lymphoid potential of the *ex vivo*-generated lymphoid progenitors. Our analysis revealed that the final cell product is heterogenous, comprising subsets with gene and protein signatures of T cell and innate lymphoid cell (ILC)/natural killer (NK) cell progenitors. We confirmed that *ex vivo*-generated lymphoid progenitors can, depending on environmental cues, further differentiate into either T cells or NK cells.

Furthermore, we identified a new role for BCL11B, a transcription factor long thought to exclusively drive T cell lineage commitment, in regulating human lymphopoiesis. These findings underscore the broad clinical potential of ProTcell products, not only for T cell reconstitution but also for generating other lymphoid lineages that can be tailored for effectively combating cancer and infections.

## Results

### *Ex vivo-*generated lymphoid progenitors recapitulate early thymopoiesis

**Figure 1:**
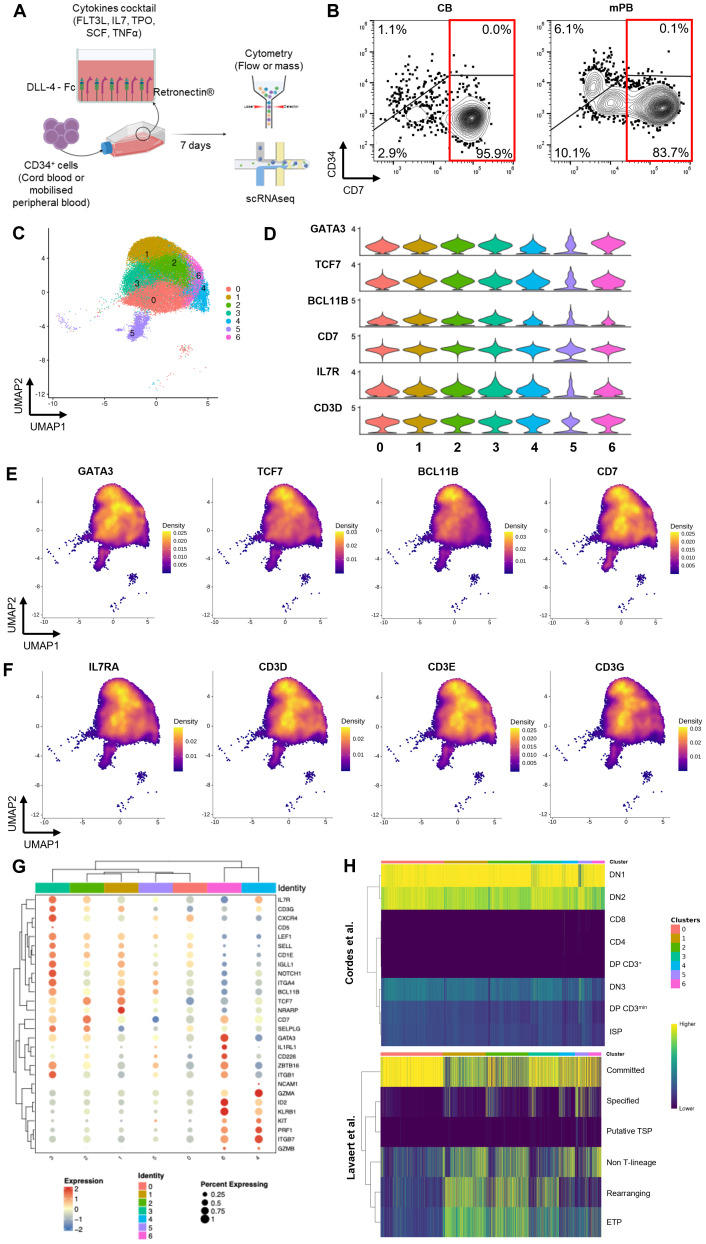
Production of ProTcell batches and their characterization at single cell level. **(A)** Experimental scheme of the ProTcell culture system: CD34^+^ cells, originated from either CB or mPB, are seeded in a culture unit coated with Retronectin and DLL4-Fc with a cocktail of cytokines in αMAM. After 7 days of culture, T cell progenitors are harvested and analyzed by flow or mass cytometry and single cell RNA sequencing. **(B)** Contour plot of flow cytometry analysis depicting the expression of CD7 and CD34 in a representative CB (right) or mPB (left) ProTcell product. **(C–H)** Further transcriptomic analyses were focused on cells positive for the CD7 transcript. **(C)** scRNAseq data was visualized by UMAP dimension reduction technique and 7 cell clusters were identified (Resolution 0.6). **(D)** Violin plots showing the normalized expression level of T lymphoid-associated genes in the 7 clusters. Density plots illustrating the gene expression of **(E)** T cell identity genes GATA3, TCF7, BCL11b and CD7, and **(F)** markers of developing T lymphoid IL7R, CD3D, CD3E and CD3G. **(G)** Clustered dot plot depicting gene expression of several markers in the 7 ProTcell clusters. Dot size indicates the percentage of cells expressing marker-specific genes in each cluster. Average expression levels of cluster-specific genes are depicted according to the color scale shown (blue represents low; red represents high). **(H)** Heatmaps illustrating the similarity scores between each individual cell from ProTcell data and annotated clusters from Cordes et al. (upper panel) and Lavaert et al. (lower panel) data. Scale bar: Z scores of the relative Spearman coefficients. Each column is representative of a single ProTcell scored across each population indicated by the row name.

To characterize the ProTcell product after a 7-day-culture on the DLL4 platform, we performed scRNAseq as well as CyTOF of the progenitors obtained from three different CB CD34^+^ and three mPB CD34^+^ starting materials (**Figure 1A**). Since both cell products are functionally similar [1], the six samples were integrated into a single scRNAseq dataset for analysis (**Supplementary Figure 1A**). Following filtering and normalization, the dataset contained 40048 cells divided into 14 clusters (**Supplementary Figure 1B**). We first analyzed the expression of CD7, an early marker of differentiating T cell progenitors, which was expressed in about 80% of the dataset’s cells (32661 cells, **Supplementary Figure 1C**).

Flow cytometric analysis confirmed that the CD7^+^ cell subset dominates the proTcell product, comprising up to 96.97% ± 0.00% of the CB-derived and 83.78% ± 0.02% for mPB-derived product after 7 days of manufacturing (**Figure 1B**), while initial CD34^+^ cells were lacking CD7 expression (**Supplementary Figure 1D**). These findings led us to identify distinct subpopulations and to characterize their potential in the final lymphoid progenitor’s product by focusing analysis on cells expressing the CD7 marker. In the scRNAseq dataset, the CD7^+^ lymphoid progenitors were clustered in seven cell subsets (**Figure 1C**). GATA3, transcription factor 7 (TCF7), and B-cell lymphoma 11B (BCL11B), the main transcriptional drivers of the T cell lineage program, were broadly expressed by these CD7^+^ cells demonstrating their commitment to the T cell lineage (**Figures 1D, E**). Additionally, interleukin 7 receptor (IL7R) and CD3 subunits, characteristic of early T cell development, were broadly expressed at the transcriptomic level (**Figures 1D, F**). It should be noted that despite the expression of CD3 subunits at transcript level, the CD3 protein was not detectable at the cell surface of the ProTcell product (**Supplementary Figure 1E**), suggesting the immature T cell characteristic of these cells. Consistent with this observation, recombination-activating gene (RAG) 1 and RAG2 transcripts were not expressed (**Supplementary Figure 1F**), supporting the absence of RAG1/2 complex-mediated T cell receptor (TCR) B gene rearrangements as demonstrated in our previous study [2].

Differential gene expression profiles of selected markers within different clusters (**Figure 1G**; **Supplementary Table 1**), as well as the top 10 genes defining each cluster (**Supplementary Figure 2**), are represented. The cells in cluster 0 correspond to earlier lymphoid commitment stage, based on their low expression of the lymphoid-associated transcription factors TCF7, BCL11B, and GATA3. In addition, these cells in cluster 0 display an enrichment of genes associated with cell cycle entry: several histone genes, cyclin-dependent kinase 1 (CDK1), proliferating cell nuclear antigen (PCNA), aurora kinase B (AURKB), and the proliferation marker Ki-67 (MKI67) (18). Cells belonging to clusters 1, 2 and 3 are instead characterized by increased expression of GATA3, BCL11B and TCF7, along with DLL4-regulated genes such as neurogenic locus notch homolog protein 1 (NOTCH1) and NOTCH regulated ankyrin repeat protein (NRARP) (**Figure 1G**; **Supplementary Table 1**). Notably, cells in cluster 3 exhibit higher expression of T cell commitment markers, including CD5, CD1E, and pre-T cell antigen receptor alpha (PTCRA), which encodes the alpha chain of the pre-TCR, suggesting they represent cells in a more advanced stage of lymphoid development (**Supplementary Figure 3A**). Supporting this hypothesis, cells in cluster 3 also demonstrate higher expression of several homing molecules—notably C-X-C chemokine receptor type 4 (CXRC4), selectin-L (SELL), and integrin alpha 4 (ITGA4)—than is expressed in other clusters (**Supplementary Figures 3B, C**).

With respect to cluster 5, differential expression analysis comparing this cluster to all others reveals that it is defined by several markers associated with proliferative activity (**Supplementary Table 1**). Specifically, this cluster shows elevated expression of key cell cycle–associated genes, including: cytoskeleton organization: cytoplasmic FMR1 interacting protein 2 (CYFIP2), rho GTPase activating protein 45 (ARHGAP45), WAS/WASL interacting protein family member 1 (WIPF1), NCK Associated Protein 1 Like (NCKAP1L) and gene expression remodeling (SWI/SNF related BAF chromatin remodeling complex subunit ATPase 2 [SMARCA2]), CCR4-NOT transcription complex subunit 1 (CNOT1) and the E3 ubiquitin ligases thyroid hormone receptor interactor 12 (TRIP12) and tripartite motif containing 56 (TRIM56) all of which are characteristic of cells actively cycling. We therefore interpret this cluster as comprising predominantly cycling cells, potentially in transitional or mixed states, rather than reflecting a lineage-restricted identity.

We then used SingleR to compare these *ex vivo*-generated ProTcells with existing datasets of *in vivo* human thymocytes (19, 20) (**Figure 1H**) and their respective transcriptional profiles. The Cordes dataset comprised early CD4-CD8- double negative (DN) thymocytes from six healthy donors. Lymphoid progenitor cells exhibit a highly similar gene expression pattern to DN1 (CD34^+^ CD38^−^ CD1a^−^) and DN2 (CD34^+/-^ CD38^+^ CD1a^−^) cell subsets of human thymocytes (**Figure 1H**) and share, to a lesser degree, the pattern of gene expression found in more advanced stages of T cell development. Additionally, ProTcells showed a higher similarity score with cells in the “committed” cluster than with those from the Lavaert dataset generated from CD34^+^ human thymocytes, while showing minimal similarity to more differentiated cells.

Taken together, these results show that the *ex vivo* generation of lymphoid progenitors from human CD34^+^ cells faithfully recapitulates the early stages of T lymphoid development.

### Expression of KLRB1 (CD161 marker) in ProTcells aligns with an ILC/NK cell gene signature

**Figure 2:**
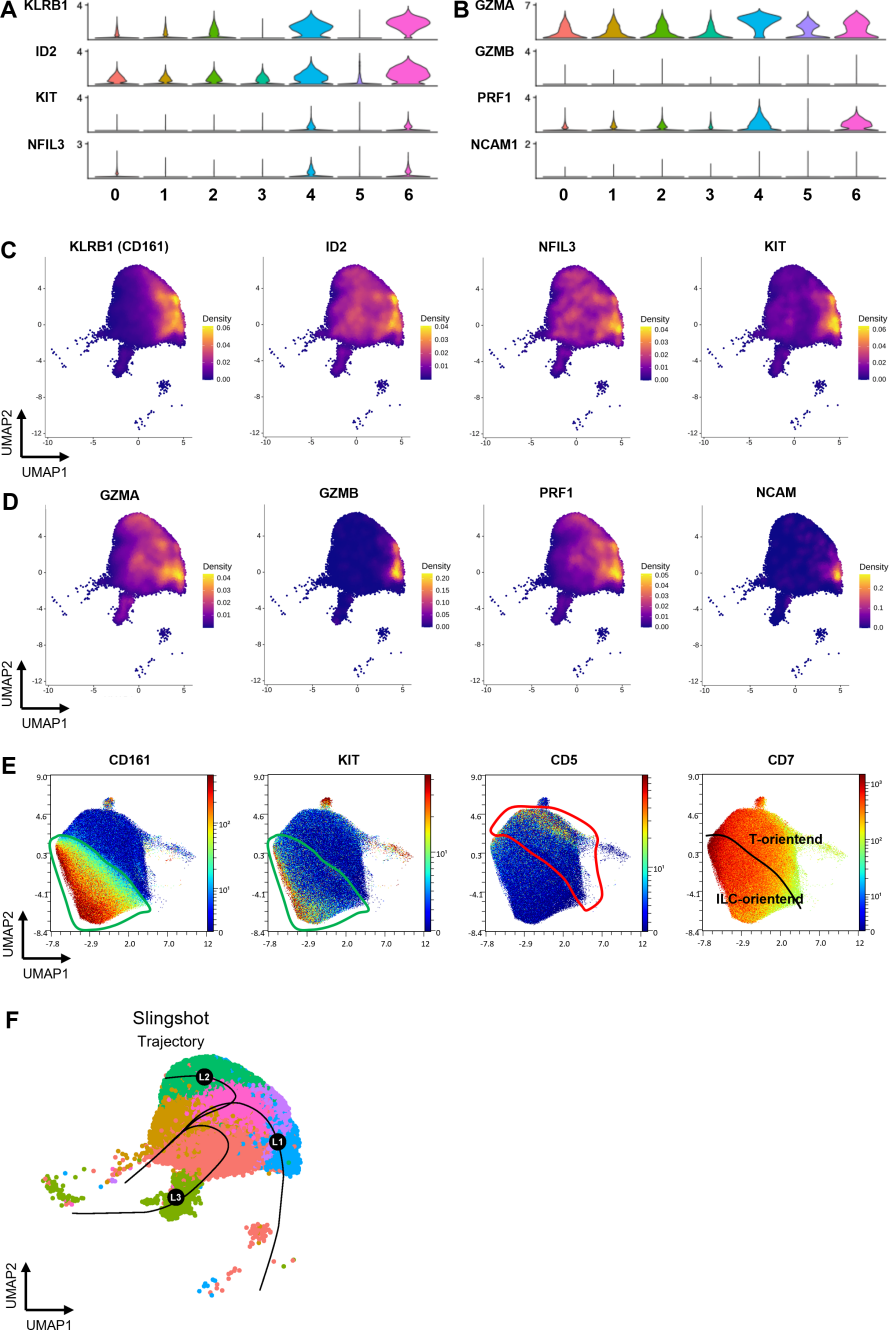
ProTcell’s subsets express innate lymphoid cell markers delineating a second cell fate. ProTcells were produced from mPB and CB CD34^+^ cells through an ex vivo system and their transcriptome (scRNAseq) or protein expression (CyTOF) were assessed in a single cell fashion, and restricted to CD7^+^ progenitors, as described in **Figures 1A, B**. Violin plots showing the normalized expression level of **(A)** ILC- and **(B)** NK-related genes in the 7 clusters. Density plots illustrating the gene expression of **(C)** the main ILC markers KLRB1, ID2, NFIL3 and KIT and **(D)** NK-related markers GZMA, GZMB, PRF1 and CD56. **(E)** CyTOF data was visualized by UMAP dimension reduction technic. UMAP projections illustrating the protein expression of CD7, CD161, KIT and CD5. The green line delineates the CD161-expressing cells along with KIT enrichment, i.e. the ILC-primed cells; the red contour excludes the CD161-expressing cells and includes CD5^+^ progenitors. **(F)** UMAP projection showing the pseudotime lineages calculated by Slingshot that describes the progressive transition along CD7^+^ clusters.

Given the presence of distinct clusters among ProTcells, we next further explored the molecular heterogeneity across these subsets. Despite expressing T cell lineage markers such as CD7, TCF7 and CD3D (**Figures 1D–F**), cells in clusters 4 and 6 were distinguished by higher expression of killer cell lectin-like receptor B1 (KLRB1, encoding for CD161), DNA-binding inhibitor 2 (ID2), nuclear factor interleukin 3 regulated (NFIL3) and the receptor tyrosine kinase (KIT) (**Figures 2A, C**) all of which are associated with ILC populations (21–23). Similarly, interleukin 1 receptor type 1 (IL1R1) and integrin beta 7 (ITGB7), prostaglandin D2 Receptor 2 (PTGDR2 or CRTH2) and CCR6, other common markers of ILC and ILC progenitors, exhibited higher expression in cells in these two clusters (**Supplementary Figure 3D, E**). Of note, GATA3 expression was higher in cluster 6 than in cluster 4 (**Figure 1D**). Conversely, cluster 4 showed stronger expression of granzyme (GZM) A, GZMB, perforin 1 (PRF1), and neural cell adhesion molecule 1 (NCAM1, encoding for CD56) (**Figures 2B, D**) than cluster 6. Since GATA3 is predominantly involved in the early stages of ILC differentiation (24, 25), while granzymes, perforin, and CD56 are associated with later stages of NK cell development, this finding suggests that cluster 4 may represent a more advanced stage of development than cluster 6.

To validate these findings, we performed CyTOF analysis, which confirmed that CD161 is expressed at the protein level in 53.4 ± 10.2% of CD7^+^ cells. This CD161^+^ cell subset contains cells that also express KIT (**Figure 2E**). This subset was, most significantly, distinct from cells exhibiting CD5 expression, which indicates commitment to the T cell lineage. In accordance with this observation, lymphoid enhancer binding factor 1 (LEF1), a T cell specific transcription factor, was shown to be expressed separately from KLRB1-positive subsets in the scRNAseq data (**Supplementary Figure 3D, F**).

This data strongly indicates that the ProTcell product is heterogenous, comprising subpopulations of lymphoid progenitors at different stages of development. These subpopulations include T cell progenitors as well as cells with a molecular profile characteristic of ILC/NK cell progenitors.

We next explored the lineage relationship between T cell and ILC/NK cell progenitors to determine whether they arise from a shared precursor population or if the DLL4 platform facilitates the expansion of two distinct lineage-biased precursors. To address this, we performed pseudotime analysis using Slingshot to delineate cell trajectories within the lymphoid cell clusters. The pseudotime analyses of CD7^+^ clusters identified three main trajectories: L1 trajectory describing the progressive transition from developing T cell subsets; L2 trajectory oriented toward clusters exhibiting an ILC/NK cell gene expression signature; and L3 trajectory oriented toward cluster 5. Of note the cluster 5 contained the cycling cells (**Figure 2F**). Differential gene expression analysis of the most significant contributors to these trajectories revealed distinct markers associated with each pathway. The L2 trajectory, leading to ILC-oriented cells (clusters 4 and 6), was enriched for markers such as GZMA, PRF1, and ITGB7 (**Supplementary Figure 4A**). In contrast, markers associated with the L1 trajectory contain components of NOTCH signaling pathway, a positive regulator of T cell development, such as NRARP and small nucleolar RNA host gene 3 (SNHG3) (26) (**Supplementary Figure 4A**). The L3 trajectory aligns with the cluster of cycling cells suggesting that this trajectory represents a lineage influenced by cell cycle dynamics and the cellular states captured along this trajectory are linked to progression through the cell cycle.

Together, these analyses suggest that the DLL4 platform allows for the expansion of a progenitor population capable of undergoing two main differentiation trajectories, one toward T cells and the other toward ILC/NK cells. Then we next aimed to functionally evaluate the lineage potential and plasticity of both T cell–oriented and NK cell–oriented subsets toward T or NK fates, *in vitro* and *in vivo*.

### KLRB1 positive and negative fractions retain T/NK cell fate plasticity

**Figure 3:**
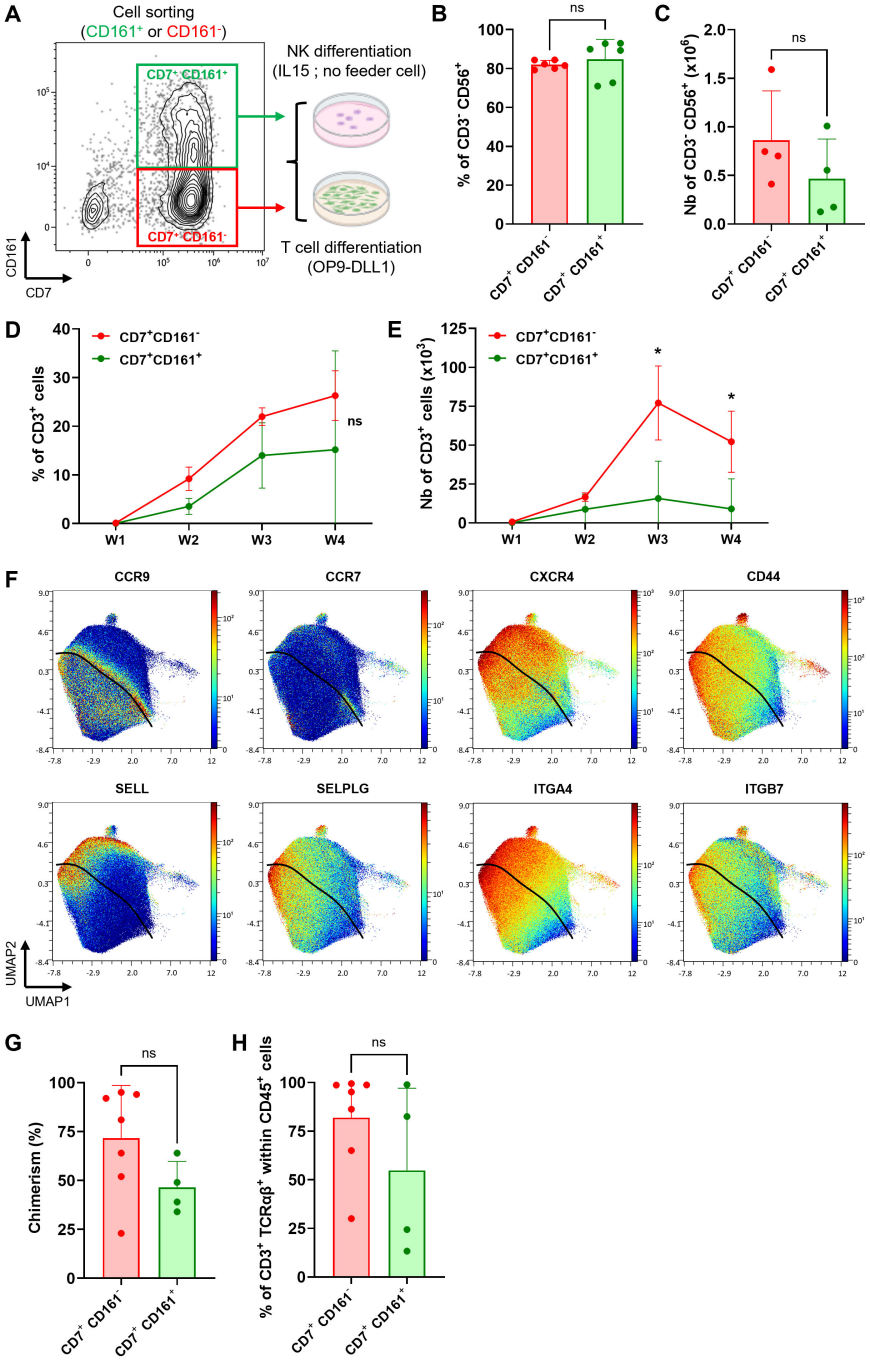
Both CD161^+^ and CD161^-^ ProTcell subsets retain T and NK cell potential as well as thymus seeding ability. **(A–E, G, H)** ProTcells were produced from CB CD34^+^ cells in 7 days as indicated in **Figure 1A** and sorted by FACS regarding their CD161 expression for further NK or T cell differentiation and functional *in vivo* experiment. To ensure the consistency, we used the same cell lot and the same sorted cell populations to perform the experiments shown in this figure, including NK cell differentiation, T cell differentiation, and the *in vivo* engraftment assay. **(A)** Experimental scheme for CD7^+^ CD161^-^ (red frame) and CD7^+^ CD161^+^ (green frame) cell subset sorting before NK or T cell differentiation. Differentiating cell phenotype was assessed by flow cytometry. **(B, C)** CD7^+^ CD161^+/-^ lymphoid progenitors were cultured for 14 days in the presence of IL15 without any feeder cell for NK differentiation. Bar plots represent the mean ± SD of **(B)** the proportion and **(C)** number of CD56^+^ NK-primed cells in the culture. **(D, E)** CD7^+^ CD161^+/-^ lymphoid progenitors were co-cultured for 4 weeks with OP9-DLL1 to advance T cell differentiation. Line plots depicting the mean ± SD of the **(D)** proportion and **(E)** number of CD3^+^ developing T cell throughout the culture. *p<0.05 values are for multiple paired Student’s t-test. **(F)** ProTcells were produced from mPB and CB CD34^+^ cells and their protein expression (CyTOF) was assessed in a single cell fashion, and restricted to CD7^+^ progenitors, as described in **Figures 1A, B**. CyTOF data was visualized by UMAP dimension reduction technic. UMAP projections illustrate the protein expression of several adhesion/homing molecules CCR9, CCR7, CXCR4, CD44, CD62L, SELPLG, ITGA4 and ITGB4. The black line separates the CD161-enriched from the CD161-devoid cells. **(G)** CD7^+^ CD161^-^ or CD7^+^ CD161^+^ cell subset has been injected separately into NSG neonates (<4-day-old). T cell reconstitution was analyzed by flow cytometry in the thymus at 6-week-post-transplantation. Bar plot representing the human chimerism (human CD45^+^ cells/human + murine CD45^+^ cells) and **(H)** the proportion of mature CD3^+^ TCRαβ^+^ T cells in the thymus of mice injected either CD161^+^ or CD161^-^ ProTcells. ns, non significant for **(B, C)** Wilcoxon signed-rank test, **(D)** multiple paired Student's t-test, **(G, H)** Mann-Whitney U test.

Our analysis of scRNAseq and CyTOF revealed a strong association between elevated CD161 expression and transcriptional commitment to the ILC/NK fate. These results are consistent with previous reports of CD161 expression during early stages of NK cell development (27–29). Furthermore, we have demonstrated the feasibility of producing a highly pure NK cell product from ProTcells using an optimized culture protocol (30). To further investigate whether NK cell potential in *ex vivo*-generated lymphoid progenitors segregated to the CD161^+^ ProTcell fraction, we used fluorescence activated cell sorting (FACS) to sort the CD7^+^ cells from the ProTcell product based on their negative or positive CD161 expression. The resulting populations were tested under either NK or T cell culture conditions (**Figure 3A**). After a 14-day-culture in NK-favoring conditions, both CD161^+^ and CD161^-^ subsets exhibited comparable capacities to differentiate into NK cells *in vitro*, as demonstrated by the presence of CD3^-^ CD56^+^ cells in similar frequencies and numbers (**Figures 3B, C**; **Supplementary Figure 5A**). In parallel, the same sorted fractions were cultured under T cell differentiation conditions using an OP9-DLL1 (OP9 cells expressing human DLL1) co-culture system for 4 weeks. Both fractions generated T cells, characterized by CD4^+^ CD8^+^ double positive (DP), CD3^+^ TCRαβ^+^, and CD3^+^ TCRγδ^+^ cell populations (**Figure 3D**; **Supplementary Figures 5B–D**). Notably, the CD161^-^ subset yielded a higher number of CD3^+^ cells (**Figure 3E**).

These results demonstrated that while CD161^+^ and CD161^-^ cell subsets display distinct gene expression pattern consistent with NK or T cell fate, respectively, both cell fractions retain lymphoid lineage plasticity. As such, both populations can differentiate *in vitro* into either T cells or NK cells, depending on the environmental cues directing their differentiation.

We then tested if cells from both CD161 subsets can colonize thymus *in vivo* upon adoptive transfer. We initially assessed the expression of surface markers involved in thymus homing and colonization using CyTOF. Both CD161^+^ and CD161^-^ subsets expressed key homing markers, including chemokine receptors (C-C chemokine receptor type [CCR] 9, CCR7, CXCR4), adhesion molecules (CD44, SELL, Selectin P Ligand [SELPLG]), and integrins (ITGA4, ITGB7) (**Figure 3F**), suggesting that both CD161^+^ and CD161^-^ cells have thymic homing ability. We next injected the same FACS-sorted CD7^+^ CD161^-^ or CD7^+^ CD161^+^ cell subsets, as used in T and NK cell differentiation assay, intra-hepatically into non-irradiated NOD.Cg-Prkdcscid Il2rgtm1Wjl/SzJ (NSG) neonate mice (**Figures 3G, H**). Six weeks post-transplantation, both fractions engrafted in the thymus, as evidenced by the presence of human CD45^+^ cells in recipient mice (**Figure 3G**). Both fractions also contributed to the development of TCRαβ^+^ CD3^+^ T cells (**Figure 3H**; **Supplementary Figure 5E**).

Overall, these results demonstrate that both CD7^+^ CD161^-^ and CD7^+^ CD161^+^ subsets retained T and NK lineage plasticity with the ability to differentiate into NK or T cells *in vitro* depending on environmental signals. Their T cell fate has been further confirmed *in vivo*, where both populations exhibit thymic homing capacity and contribute to thymopoiesis.

### BCL11B expression does not suppress the NK potential in lymphoid progenitors

The dual potential of ProTcells raises important questions about the molecular mechanisms governing lineage specification in early human lymphoid progenitors. The specification toward either the T or ILC lineage is tightly regulated by opposing transcription factors (31): commitment to one lineage requires the repression of the alternative gene program. In this context, we focused on BCL11B, a transcription factor traditionally viewed as a master regulator of T cell development and an inhibitor of NK/ILC differentiation (32–34). However, the role of BCL11B in regulating NK/ILC differentiation is controversial as other studies have shown that it is essential for the development and function of ILCs in mice (35, 36). Our data indicates that BCL11B is widely expressed in CD7^+^ lymphoid progenitors (**Figures 1D, E**), including in CD161^+^ ILC/NK-fated cells, albeit at lower level (**Supplementary Figure 4B**). This finding suggests that BCL11B expression does not entirely abrogate ILC/NK potential in human CD7^+^ lymphoid progenitors.

**Figure 4:**
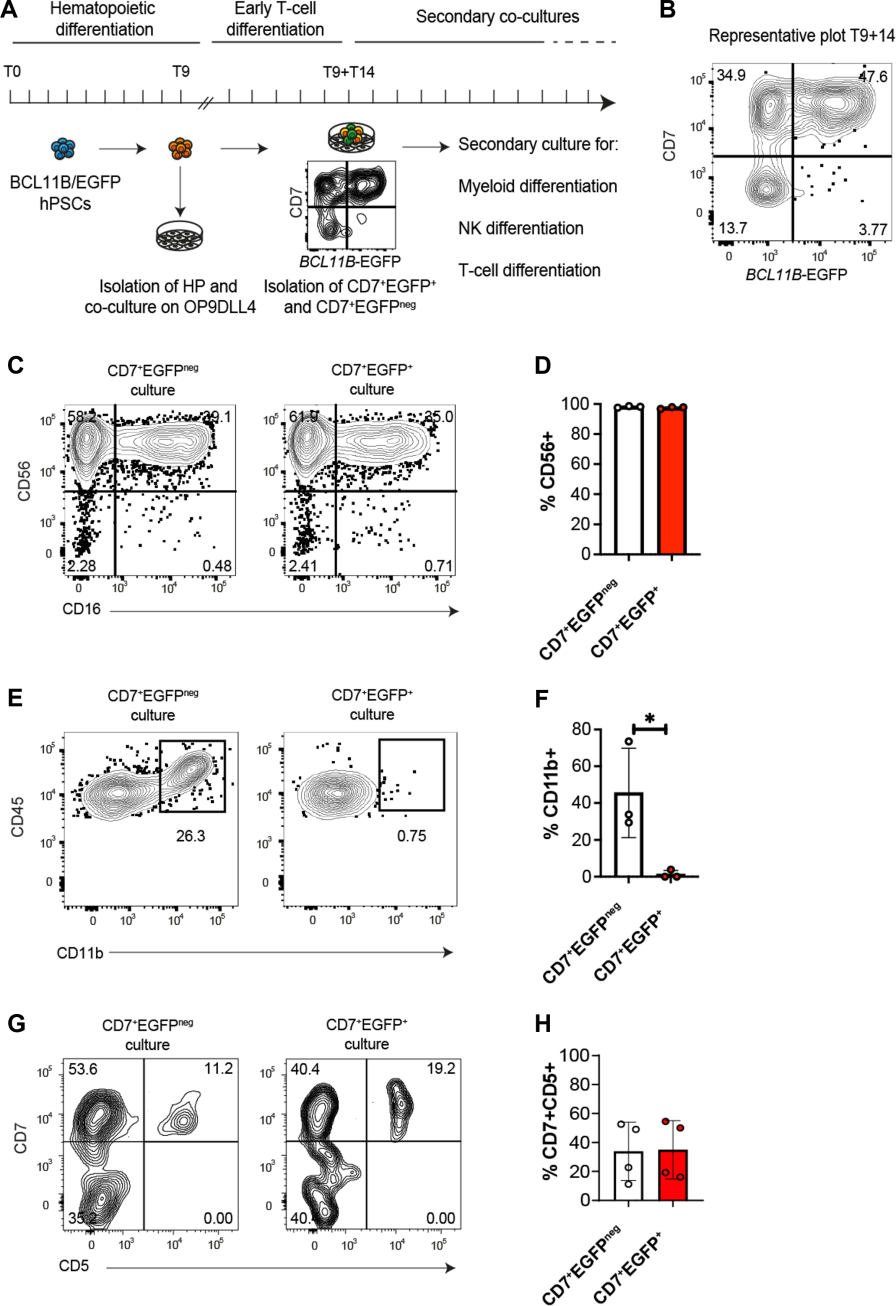
Activation of BCL11B regulatory elements does not restrict NK cell potential. **(A)** Experimental design for assessing BCL11B role in human T cell commitment. EGFP-BAC reporter was integrated at exon 1 of endogenous *BCL11B* gene in hPSC, resulting in a monoallelic disruption of this gene and the creation of *BCL11B*-EGFP reporter cell line. *BCL11B*-EGFP hPSCs were differentiated into hematopoietic progenitors (HP) for 9 days. The generated HP cells were isolated for early T cell differentiation on OP9-DLL4 for 14 more days. At this time, T9+T14, *BCL11B*-EGFP negative and positive progenitor cells had been sorted by FACS and secondary cultures were performed to assess their myeloid, NK and T cell potential. Phenotypes were assessed at each stage by flow cytometry for myeloid, lymphoid, NK or T membrane markers. **(B)** Representative dot plot of flow cytometry analysis of *BCL11B*-EGFP hPSC-derived progenitors upon sorting at day T9+T14 after culture on OP9-DLL4 for 14 days. At this stage, CD45^+^ CD56^-^ CD7^+^ cells are clearly distributed into two populations, according to *BCL11B*-EGFP expression. **(C, D)** *BCL11B-EGFP* ^neg/+^ progenitors were co-cultured for 10 days with OP9-DLL4 with IL15 for NK differentiation. **(C)** Representative contour plots of flow cytometry analysis illustrating the expression of CD56, delineating NK differentiation, and CD16, translating NK maturation. **(D)** Bar plot representing the mean ± SD of CD56-expressing NK-primed cells in three independent experiments. *p<0.05 values are for paired Student’s t-test. **(E, F)** *BCL11B*-EGFP^neg/+^ progenitors were co-cultured 5 days with OP9 for myeloid differentiation. **(E)** Representative contour plots of flow cytometry analysis illustrating CD11b expression translating myeloid priming. **(F)** Bar plot representing the mean ± SD of CD11b-expressing myeloid progenitors in three independent experiments. *p<0.05 values are for paired Student’s t-test. **(G, H)** *BCL11B*-EGFP^neg/+^ progenitors were co-cultured 7 days with OP9-DLL4 devoid of IL15 for T cell differentiation. **(G)** Representative contour plots of flow cytometry analysis illustrating the expression of CD7 and CD5 translating T cell differentiation. **(F)** Bar plot representing the mean ± SD of CD7 and CD5-expressing T cell progenitor cells in four independent experiments.

To further investigate the specific role of BCL11B in human lymphoid specification, we used a knock-in *BCL11B*-Enhanced green fluorescent proteins (EGFP) reporter human pluripotent stem cell (hPSC)-line with a bacterial artificial chromosome (BAC)-based monoallelic integration of the EGFP transgene within the endogenous exon 1 of *BCL11B* gene (**Supplementary Figure 6A**). Differentiating this cell line into T cell progenitors allowed us to assess the T, NK and myeloid potential of either *BCL11B*-EGFP^+^ or *BCL11B*-EGFP^neg^ hPSC-derived T cell progenitors (**Figure 4A**). We isolated CD34^+^ CD144^+^ CD43^-^ CD73^-^ CXCR4^-^ hematopoietic precursors from embryoid body-based differentiation cultures (37) and we cultured them on OP9-DLL4 for 14 days in T cell specification medium, enabling the emergence of CD7^+^ progenitors (**Figures 4A, B**). These CD45^+^ CD56^-^ CD7^+^ hematopoietic progenitors were FACS-sorted into *BCL11B*-EGFP^+^ and *BCL11B*-EGFP^neg^ fractions and further cultured on either OP9-DLL4 or OP9 to assess their T/NK lymphoid and myeloid potential, respectively.

In OP9-DLL4-based NK cell potential assay, both *BCL11B*-EGFP^+^ and *BCL11B*-EGFP^neg^ fractions generated CD45^+^ CD56^+^ NK lymphoid cells (**Figures 4C, D**). In contrast, only *BCL11B*-EGFP^−^ progenitors could generate CD45^+^ CD11b^+^ myeloid cells (**Figures 4E, F**), which were EGFP negative at the time of analysis (**Supplementary Figures 6B, C**). Thus, while the activation of BCL11B regulatory elements in CD7^+^ progenitors does not preclude NK cell differentiation, it marks the stage where myeloid potential is repressed.

When T cell potential was tested on OP9-DLL4 co-cultures, both *BCL11B*-EGFP^+^ and *BCL11B*-EGFP^neg^ progenitors upregulated CD5, a marker associated with T cell developmental progression (**Figures 4G, H**). *BCL11B*-EGFP expression was also dynamically regulated in T cell cultures, with both sorted progenitor subsets yielding a mixture of EGFP^neg^ and EGFP^+^ cells (**Supplementary Figure 6D**). Significantly, CD4^+^ CD8^+^ DP cells were rarely generated using the *BCL11B*-EGFP reporter line, likely due to the BCL11B heterozygosity caused by the monoallelic reporter integration that mimics BCL11B haploinsufficiency (38–40) (not shown).

As an overall conclusion, despite the expression of BCL11B, the ProTcell platform stands at the intersection of the NK/ILC and T cell lymphoid lineages. In summary, our experimental model with human hematopoietic progenitors shows that the BCL11B regulatory element is activated early in lymphoid commitment, specifically in cells harboring both T and NK potential that lost the ability to generate myeloid cells.

## Discussion

We have previously shown that our DLL4-based culture system is compatible with both CB- and mPB-derived HSPCs and demonstrated the homogeneity of the final cell product despite the different initial material sources (16). In the present study, we combined six different samples from two HSPC sources and performed an in-depth characterization of our *ex vivo*-generated lymphoid progenitors. To capture the heterogeneity of the ProTcell product, we performed scRNAseq and CyTOF analyses which revealed a strong induction of T cell-related transcription factors TCF7, BCL11B and GATA3, which are known to be upregulated upon NOTCH signaling (41–45), as well as for their early development of T cell markers such as CD7 and IL7R. Comparative analysis with *in vivo* thymocytes revealed strong similarities between these cells and the most immature lymphoid progenitors found in the human thymus. Furthermore, the absence of transcripts for the RAG1/2 complex, crucial for TCR gene rearrangement, aligns with prior findings on the absence of TCR rearrangement (13), confirming the ProTcells’ immature and early developmental stage status.

ScRNAseq analysis also showed that the *ex vivo*-generated cell product is inherently heterogeneous, comprising distinct cell subset clusters identified through differential gene expression. Integrated bioinformatic analysis highlighted the presence of a lineage trajectory leading to the initiation of an ILC/NK gene program in a subset of cells. The ProTcell product includes a significant proportion of cells expressing the early NK differentiation marker CD161 and genes frequently associated with ILC/NK progenitors, such as IL7R, ITGB7, IL1R1, ID2, NFIL3, KIT, PTGDR2 and CCR6 (22, 46–51), but our work highlights that these cells remain plastic, as they can generate T cells both *in vitro* and *in vivo.* Notably, due to our model’s non-permissive background for human NK cell development, we observed no NK cells in our *in vivo* studies (52). Investigating NK potential *in vivo* will require alternative models, such as human interleukin (IL)15 transgenic mice (53). While our analysis focused on differentiated cells at day 7, we acknowledge that complementary scRNAseq and mass cytometry profiling of freshly isolated CD34^+^ cells at day 0 would provide valuable baseline data and further contextualize the differentiation dynamics observed. This remains an important avenue for future investigation.

The presence of cells with distinct differentiation trajectories in the ProTcell product offers an opportunity to investigate what regulates lymphoid lineage fate decisions. In this study, we focused on BCL11B, a Krüppel-like C2H2-type zinc finger transcription factor (54). The role of BCL11B in lineage differentiation is multifaceted and somewhat controversial. Studies using knockout mouse models (in which the mice die shortly after birth) have revealed BCL11B’s essential role in the development of αβ T cells and neuronal corticospinal motor neurons (55, 56). While BCL11B clearly restricts T cell progenitors from adopting an ILC fate through ID2 repression, NK cell development remains intact in BCL11B-deficient mouse models (33). In humans, heterozygous mutations in BCL11B have been linked to intellectual disability and varying degrees of immunodeficiency (39, 40). Furthermore, clinical evidence suggests that BCL11B promotes human NK cell differentiation, as patients with heterozygous BCL11B mutations display impaired early NK cell maturation (38). This evidence suggests a species-specific role for this transcription factor.

In this study, we show that BCL11B is expressed early in CD7^+^ progenitors and that cells with active BCL11B regulatory elements harbor a dual T/NK potential. These findings align with previous reports that identify BCL11B as part of a regulatory network directly promoting NK cell differentiation (38). Further work is needed to elucidate whether and how BCL11B directly regulates the myeloid differentiation program. Understanding the precise mechanisms underlying BCL11B’s regulation of lineage specification will provide critical insights into both normal hematopoiesis and immune dysfunctions associated with BCL11B mutations.

Given that thymopoiesis and ILC differentiation are mostly described as occurring in separate locations, finding a dual T/ILC potential was initially unexpected. However, several works have documented the presence of progenitors in the thymus that retain the ability to generate cell types other than T cells, including dendritic cells (DC), plasmacytoid DCs, NK cells, ILCs, and, to a lesser extent, B cells (19, 20, 57, 58). Additionally, recent reviews have compiled information about the presence of NK and ILCs in the thymus and prompted a renewed consideration of their possible maturation in the thymus (49, 59–64). Despite the identification of progenitors with restricted potential toward T or ILC fates in humans (50, 65, 66), the presence of multipotent or at least double T/ILC potent common progenitors in the thymus or in circulation is plausible (47, 66, 67).

Moreover, recent reports have described ILCs expressing TCR transcripts, with ILC type 2 and NK cells specifically exhibiting unfunctional rearranged TCR loci (68–70). These observations support the hypothesis that ILC differentiation may originate from a branching pathway of aborted DN thymocytes (64). Considering *ex vivo*-generated progenitors as an artificial counterpart to DN thymocytes, it is reasonable to infer that they may retain potential toward the ILC lineage (71). Notably, Notch signaling, which is the foundation of our *ex vivo* culture system, plays a crucial role in the maturation, functionality, and plasticity of ILCs, though it is not essential for their early specification (24, 66, 72–76). In line with this, it has been proposed that some human NK cells expressing intracellular CD3 are generated through Notch signaling during their early development (77). Indeed, the strength and duration of Notch signaling influence the lineage fate of progenitors between T and ILC lineages (24, 66, 78). Strong and sustained signaling drives T cell and ILC3/LTi development (42, 73, 79), while weaker or transient signaling favors NK/ILC1 and ILC2 fates (70). In our system, although DLL4 is present, Notch activation may vary between cells due to differences in receptor expression, contact with stromal cells, or culture density. This heterogeneity likely allows some progenitors to receive suboptimal signaling and adopt non-T cell fates. This hypothesis is supported by findings from OP9-DLL1 co-cultures, where similar variability in Notch signaling permits the emergence of diverse ILC subsets under defined cytokine conditions (80). Thus, although constitutive Notch signaling biases progenitors toward T cell differentiation, it does not entirely inhibit ILC commitment, as progenitors can still develop into ILCs under permissive conditions (46, 80). Our clinical trials will play a crucial role in monitoring the full cellular potential of the therapeutic cell product, with patient follow-ups providing insights into the reconstitution of ILC/NK cells and conventional T cells. Since these immune cell types serve distinct but complementary roles in human immunity, their combined application could enhance efficacy and improve the outcomes of future therapeutic strategies.

## Materials and methods

### Human samples

Umbilical cord blood (CB) samples were collected from donors at AP-HP, Saint Louis Hospital (*Unité de Thérapie Cellulaire, CRB-Banque de Sang de Cordon, Paris, France*) in accordance with ethically approved protocols and following the provision of informed consent. CD34^+^ HSPCs were magnetically enriched (purity > 90%) from CB as described previously (81). For specific experiments, enriched CD34^+^ cells were purchased from Lymphobank (Besançon, France) and Tebu-bio (Le Perray-en-Yvelines, France).

### ProTcell production

For ProTcells’ culture, human CD34^+^ HSPCs isolated either from CB or mPB were cultured for 7 days at a cell concentration of 5x10^4^ and 1x10^5^ cells/mL respectively with 5µg/mL human DLL4 fused to IgG Fc fragment (DLL4-Fc) protein and 2 µg/mL RetroNectin^®^ (Takara Bio Europe, Saint-Germain-en-Laye, France)-coated wells of culture plates/culture flasks containing α minimal essential medium (MEM) (Gibco, Life Technologies, Courtaboeuf, France) supplemented with 20% defined fetal bovine serum (FBS) (HyClone, Cytiva Europe GmbH, Velizy-Villacoublay, France) and human cytokines (100ng/mL IL7, 100ng/mL FMS-like tyrosine kinase 3 ligand [Flt3L], 100ng/mL stem cell factor [SCF] and 100ng/mL thrombopoietin [TPO]; all from PeproTech France, Neuilly-sur-Seine, France. Also used was 100ng/mL TNFα (R&D Systems, Minneapolis, MN). Medium was half-changed after 3 days of culture (13).

### Feeder-free NK cell differentiation

The *in vitro* NK cell potential of FACS-sorted CD7^+^ CD161^-^ and CD7^+^ CD161^+^ progenitors was assessed through an IL15-based feeder-free NK cell differentiation assay. The CD7^+^ CD161^-^ and CD7^+^ CD161^+^ progenitors sorted from CB ProTcells were cultivated in RPMI 1640 Medium, GlutaMAX™ Supplement (Gibco, Life Technologies, Courtaboeuf, France) supplemented with 10% defined FBS (HyClone, Cytiva Europe GmbH, Velizy-Villacoublay, France) and human cytokines (20ng/mL IL7, 50ng/mL Flt3L, 50ng/mL SCF, 20ng/mL IL15, 500 IU/mL IL2; all from PeproTech Inc., Rocky Hill, NJ). Medium was refreshed every 2–3 days. After 1 and 2 weeks of culture, cells were harvested and analyzed (using flow cytometry) for the presence of CD56^+^ NK cells.

### *In vitro* T cell differentiation assay

The *in vitro* T lymphoid potential of FACS-sorted CD7^+^ CD161^-^ and CD7^+^ CD161^+^ progenitors was assessed by using an OP9-DLL1 co-culture system, as described previously (81). CD7^+^ CD161^-^ and CD7^+^ CD161^+^ progenitors sorted from CB ProTcells were co-cultured on OP9-DLL1 stromal cells for 4 weeks in αMEM (Gibco, Life Technologies, Courtaboeuf, France) supplemented with 20% defined FBS (HyClone, Cytiva Europe GmbH, Velizy-Villacoublay, France), and human cytokines (2ng/ml IL7, 5ng/ml Flt3L, 10ng/mL SCF and 10ng/mL TPO, all from PeproTech France, Neuilly-sur-Seine, France). Every week, a part of the co-cultured cells were harvested and analyzed (using flow cytometry) for the presence of CD4^+^ CD8^+^ and TCRαβ- or TCRγδ-expressing CD3^+^ T cells, and the other part of the co-cultured cells were reseeded on fresh OP9-DLL1 cells.

### Generation of reporter bacterial artificial chromosome

The BAC plasmid clone CH17-365C4 containing the *BCL11B* gene flanked by approximately 90kb upstream and 36kb downstream of the start codon was obtained from CHORI BACPAC (Children ‘s Hospital Oakland Research Institute, Oakland, CA, USA). To insert an EGFP reporter cassette into the *BCL11B* locus of the BAC plasmid, an EGFPpA-LoxP-PGK-Gb2-Kan/Neo-pA-LoxP vector was targeted to the start codon of the *BCL11B* gene using recombination-mediated genetic engineering. In brief, DH10b bacteria, containing the CH17-365C4BAC plasmid were grown under chloramphenicol antibiotic selection (Sigma-Aldrich). pSC101BAD Gba A[tet] (Genebridges, Heidelberg, Germany) was transfected into these BAC-containing bacteria and grown at 32°C under tetracyclin antibiotic resistance (Sigma-Aldrich). Bacterial cells containing both plasmids were then transfected with PCR amplified targeting construct (EGFPpA-LoxP-PGK-Gb2-Kan/Neo-pA-LoxP), using primers with overhanging 50bp homology arms, and grown overnight at 37°C under chloramphenicol and kanamycin resistance (Sigma-Aldrich). BAC DNA from selected bacteria was purified and verified for correct integration of the EGFP cassette using restriction analyis. The *BCL11B*-EGFP reporter construct was subsequently functionally validated by transfection in T cell leukemia Jurkat cells, which express levels of BCL11B within a biologically relevant range, similar to BCL11B expression during human embryonic stem cell line (hESC)-derived T cell differentiation. The erythromyeloid leukemia line K562 cell was used as negative control. After transfection and neomycin selection, the selection cassette was removed using Cre recombinase and expressed EGFP as expected.

### Generation of reporter hESC

A total of 2x10^6^ single cell adapted hESC were nucleofected with 5μg purified BAC reporter using Lonza Amaxa human stem cell nucleofector kit 2 (Lonza) with program F16. Cells were replated at high density (1x10^5^ cells/cm^2^) in single cell conditions on drug resistant DR4 mouse embryonic fibroblasts (MEF). A total of 10μM of the Rock inhibitor Y-27632 (Selleckchem) was added. G418 (50μg/ml) (Gibco) was added from day three onwards and retained for three weeks. Single colonies were picked, expanded and screened for transgene integration. Colonies showing transgene integration were transfected using pCAGGS-NLS-Cre-PGK-Puro and kept under puromycin (Sigma-Aldrich) selective pressure (300ng/ml) for three days to remove the neomycin selection cassette. Single colonies were expanded and screened by PCR. Genomic DNA was isolated using Genelute mammalian genomic DNA miniprep kit (Sigma-Aldrich) according to the manufacturer’s instructions. Colonies containing successfully floxed cells were clonally expanded using single cell deposition on the FACS Aria. (clone 3.8 and 3.10). To determine the integration site of the BAC, targeted locus amplification (TLA) sequencing was performed which revealed homologous integration of the EGFP reporter gene in the endogenous *BCL11B* locus in one allele (Cergentis). No random integration at other genomic sites was detected.

### Human pluripotent stem cells

*BCL11B*-EGFP human embryonic stem cell line (hESC) was cultured on irradiated MEF, in Dulbecco’s Modified Eagle medium (DMEM)/F12 medium (Corning) supplemented with 20% of KnockOut™ Serum Replacement (Thermo Fisher Scientific), 1% penicillin-streptomycin (Lonza), 1% glutamine (Lonza), 0.1% 2-Mercaptoethanol (Sigma-Aldrich) and 0.7% of MEM Non-Essential Amino Acids Solution (Thermo Fisher Scientific). Basic fibroblast growth factor (bFGF) at 20ng/mL (Miltenyi Biotech) was added to the hESC medium shortly before use.

### Hematopoietic differentiation protocol

hESCs were expanded for two passages on MEF and one passage on Matrigel (Corning), for feeder-depletion. After 24–48 hours on Matrigel, cells were dissociated by TripLE treatment (Thermo Fisher Scientific) plus scraping and collected in “wash medium” (DMEM/Hams F-12 [Corining] 50/50 mix, 5% KnockOut™ Serum Replacement [Thermo Fisher Scientific] and 25mM HCl) supplemented with 10µg/mL DNAse I (Calbiochem, 260913). At day 0, cells were resuspended as embryoid bodies (EB) at a maximum density of 250000 cells/mL and plated into non-adherent 6-wells plates coated with 5% polyheme solution (Sigma). The differentiation medium was supplemented as previously described (82). The base medium for the first three days of mesoderm induction was Serum-Free Differentiation (SFD) medium (IMDM/Ham’s F-12 [Corning] 75/25 mix with 0.05% w/v bovine serum albumin (BSA) [Sigma-Aldrich]). On the first day of differentiation, SFD medium was supplemented with 2mM l-glutamine (Lonza), 1mM ascorbic acid (Sigma-Aldrich), 400µM 1-thioglycerol solution (MTG, Sigma-Aldrich), 150µg/mL transferrin (R&D System) and 10ng/mL bone morphogenetic protein 4 (BMP4, R&D System). Twenty-four hours later, 5ng/mL bFGF (R&D System) was added. At the second day of differentiation, 3µM CHIR99021 (Cayman Chemical Company) was added. On the fourth day, EBs were changed to StemPro-34 medium (Thermo Fisher Scientific) supplemented with penicillin–streptomycin, l-glutamine, ascorbic acid, 1-thioglycerol and transferrin, as above, with additional 5ng/mL bFGF and 15ng/mL vascular endothelial growth factor (VEGF, R&D). On day 6, 10ng/mL IL6, 25ng/mL insulin-like growth factor 1 (IGF1), 5ng/mL IL11, 50ng/mL SCF (all from Miltenyi Biotech) and 2U/mL erythropoietin (EPO) (Peprotech) were added.

### Myeloid, T cell and NK-differentiation from hPSC-derived precursors

For differentiating hematopoietic precursors into BCL11B-expressing hematopoietic progenitors, CD34^+^ CD144^+^ CD43^-^ CD73^-^ CXCR4^-^ cells were FACS-sorted from day 9 EB-culture and plated on confluent OP9-DLL4-coated 24-multiwell plates. Differentiating cells were cultured in αMEM (Thermo Fisher Scientific) with 2.2g/L sodium bicarbonate (Corning), supplemented with 20% FBS (HyClone), 1% penicillin - streptomycin (Lonza), 1% glutamine (Lonza) and 400µM MTG (Sigma-Aldrich). Base medium was enriched with 50ng/mL SCF (only for the first week), 5ng/mL Flt3L and 5ng/mL IL7 (all from Miltenyi Biotech) to engage T cell lineage specification. At day 14, both *BCL11B*-EGFP positive and negative fractions were FACS-sorted from CD45^+^ CD56^-^ CD7^+^ hematopoietic progenitors and further cultured on either OP9 or OP9-DLL4 to assess their myeloid and T/NK lymphoid potential. Myeloid differentiation was induced after 5–6 days on OP9 stroma, by using the above-mentioned αMEM base medium supplemented with 50ng/mL SCF, 50ng/mL macrophage colony stimulating factor (M-CSF), 10ng/mL IL3, 10ng/mL Flt3L and 30ng/mL granulocyte-macrophage colony-stimulating factor (GM-CSF) (all from Miltenyi Biotech). NK-specific differentiation was performed by leaving hematopoietic progenitors for an additional 10–14 days on OP9-DLL4 with the above described “T cell medium” supplemented with 10ng/mL IL15 (Miltenyi Biotech). For T cell specification, a medium with the same composition as above was used, and the culture was split every 4–5 days and replate onto new stroma.

### Flow cytometry

For surface staining, cells were incubated with the appropriate antibodies, listed in **Table 1**, for 15-30 min on ice, washed and then resuspended in staining buffer (phosphate buffered saline [PBS, EurobioScientific, France] supplemented with 0.5% w/v BSA [EurobioScientific, France], 2mM Ethylenediaminetetraacetic acid [EDTA, Invitrogen, Life Technologies, Courtaboeuf, France]).

**Table 1:** Antibodies used for flow cytometry phenotyping.

Antibody	Fluorochrome	Supplier	Clone	Cat#
hCD1a	BV510	BD Pharmingen	HI149	563481
hCD11c	APC	BD Pharmingen	B-ly6	559877
hCD14	BV510	Sony	M5E2	2109210
hCD15	FITC	BD Pharmingen	MMA	332778
hCD16	VioBlue	Miltenyi Biotec	REA423	130-113-396
hCD161	FITC	Miltenyi	191B8	130-113-592
hCD19	BV510	Sony	HIB19	2111210
hCD3	BV421	Sony Biotechnology	UCHT1	2102170
hCD3	PE-Cy7	eBioscience	UCHT1	25.0038.42
hCD33	PE	BD Pharmingen	WM53	555450
hCD34	PE-Cy7	Sony	581	2317580
hCD34	APC	Miltenyi	AC136	130-113-176
hCD34	BV421	BD Horizon	581	562577
hCD38	PE	BD Biosciences	HIT2	555460
hCD4	APCVio770	Miltenyi Biotec	REA623	130-109-454
hCD45	BV510	Sony Biotechonology	HI30	2120180
hCD45	PerCP-Cy5.5	Biolegend	HI30	304027
hCD5	PE	BD Pharmingen	UCHT2	555353
hCD5	PE-Cy7	BioLegend	UCHT2	300622
hCD56	APCVio770	Miltenyi Biotec	REA196	130-114-548
hCD7	FITC	BD Pharmingen	M-T701	555360
hCD7	PE-Vio770	Miltenyi	CD7-6B7	130-123-890
hCD8	PEVio770	Miltenyi Biotec	REA734	130-110-680
hNKp30	APC	Miltenyi Biotec	REA823	130-112-430
hNKp44	PEVio770	Miltenyi Biotec	REA1163	130-120-359
hNKp46	PE	BD	9E2/NKp46	557991
mCD45	APC-Cy7	Biolegend	30-F11	103115
Pan TCRαβ	APC	BioLegend	IP26	306718
Pan TCRγδ	PE	Miltenyi Biotec	11F2	130-113-504

All flow cytometry data were acquired with a MACSQuant^®^ analyzer (Miltenyi Biotech) or a Gallios flow cytometer (Beckman Coulter, Krefeld, Germany) and then analyzed using FlowJo software (version 10.10.0, TreeStar, Ashland, OR, United States) or Kaluza Analysis Software (version 2.3, Beckman Coulter, Krefeld, Germany). During flow cytometry analyses, all gatings were performed on live cells (determined by exclusion of the dye 7AAD [BD biosciences, Le Pont-de-Claix, France]). Results are shown in 2D dotplots and populations are represented by contour plot with outliers.

### Cell sorting

For surface staining, cells were incubated with the appropriate antibodies for 30 min on ice, washed and then resuspended to 2x10^7^ cells/mL in PBS (EurobioScientific, France) 2% FBS (HyClone, Cytiva Europe GmbH, Velizy-Villacoublay, France), supplemented with 50µg/mL Gentamicin (Gibco, Life Technologies, Courtaboeuf, France).

A FACSAria II SORP cell sorter (BD Biosciences) in the two-way high purity mode was used for this sorting. Cells were recovered in αMEM (Gibco, Life Technologies, Courtaboeuf, France) supplemented with 20% defined FBS (HyClone, Cytiva Europe GmbH, Velizy-Villacoublay, France) and 50µg/mL Gentamicin (Gibco, Life Technologies, Courtaboeuf, France).

### Cell staining for mass spectrometry (CyTOF)

A large-scale mass cytometry analysis was performed using phenotypic and functional markers, listed in **Table 2**, allowing the identification of populations and subpopulations within samples.

**Table 2:** The antibody panel used with mass cytometry for ProTell characterization.

Isotope	Antibody	Supplier	Clone	Cat#
089Y	CD45	Fluidigm	HI30	3089003C
106Cd	CD90	BioLegend	5E10	328102
110Cd	CD14	BioLegend	M5E2	301802
111Cd	CD53	BD	HI29	555506
113Cd	CD2	BioLegend	RPA-2.10	300202
114Cd	CD244	BioLegend	2-69	393502
116Cd	CD4	BioLegend	SK3	344602
141Pr	CD3	Fluidigm	UCHT1	3141019C
142Nd	CD19	Fluidigm	HIB19	3142001C
143Nd	CD5	Fluidigm	UCHT2	3143007C
144Nd	CD69	Fluidigm	FN50	3144018C
145Nd	CD7	Fluidigm	CD7-6B7	3145013C
146Nd	CD64	Fluidigm	10.1	3146006C
147Sm	CD11c	Fluidigm	Bu15	3147008C
148Nd	CD110	BioLegend	S16017A	393802
149Sm	CD25 (IL2R)	Fluidigm	2A3	3149010C
150Nd	CD164	BioLegend	67D2	324802
151Eu	CD123	Fluidigm	6H6	3151001C
152Sm	CD120b	BioLegend	3G7A02	358402
153Eu	CD62L (SELL)	Fluidigm	DREG-56	3153004C
154Sm	CD133	BioLegend	S16016B	394002
155Gd	CD117 (KIT)	BioLegend	104D2	313202
156Gd	CD10	Fluidigm	HI10a	3156001C
158Gd	CD135 (Flt3)	Fluidigm	BV10A4H2	3158019C
159Tb	CD197	Fluidigm	G043H7	3159003A
160Gd	CD120a	BioLegend	W15099A	369902
161Dy	CD162	Fluidigm	KPL-1	3161026C
162Dy	ITGB7	Fluidigm	FIB504	3162026C
163Dy	CD34	Fluidigm	581	3163014C
164Dy	CD161	Fluidigm	HP-3G10	3164009C
165Ho	CD127 (IL7R)	Fluidigm	A019D5	3165008C
166Er	CD74	Fluidigm	LN2	3166018C
167Er	CD1a	Fluidigm	HI149	3167012C
168Er	CD199 (CCR9)	Fluidigm	L053E8	3168011C
169Tm	CD33	Fluidigm	WM53	3169010C
170Er	CD45RA	Fluidigm	HI100	3170010C
171Yb	CD44	Fluidigm	IM7	3171003C
172Yb	CD38	Fluidigm	HIT2	3172007C
173Yb	CXCR4	Fluidigm	12G5	3173001C
174Yb	CD49d	Fluidigm	9F10	3174018C
175Lu	CD71	Fluidigm	OKT-9	3175011C
176Yb	CD56	Fluidigm	NCAM16.2	3176008C
209Bi	CD11b (Mac-1)	Fluidigm	ICRF44	3209003C

Labelled antibodies were supplied by Fluidigm, Inc Canada, except for CD2, CD4, CD14, CD90 CD110, CD117, CD120a, CD120b, CD133, CD164, CD244 (BioLegend) and CD53 (BD Bioscience). The latter were labelled manually using Maxpar X8 Antibody Labeling Kit for Lanthanides and Maxpar MCP9 Antibody Labeling Kit for Cadmium (Fluidigm, Inc Canada), following manufacturer’s instructions.

Each antibody was functionally validated and titrated in a mix of cells containing peripheral blood mononuclear cell (PBMC), CD34^+^ HSPCs and ProTcells.

Cells stored in liquid nitrogen were thawed in pre-warmed RPMI 1640 Media + GlutaMAX™ (Gibco, Invitrogen, France) and washed in PBS (EurobioScientific, France). Cells were incubated in PBS with Cisplatine Cell-ID™ (Fluidigm, Inc Canada) at 2.5µM for 5 minutes at room temperature (RT) for viability staining. Cells were then washed using a staining solution (PBS 0.5% w/v BSA [EurobioScientific, France], 2mM EDTA [Invitrogen, Life Technologies, Courtaboeuf, France]) and resuspend in 50µL. Staining mix was added on cells during 30 minutes at RT. Following staining, cells were washed with staining solution and fixed with 300µL of staining solution 2% paraformaldehyde (Sigma-Aldrich, Lyon, France) for 15 minutes at RT.

For intracellular staining, cells were permeabilized using Foxp3/Transcription Factor Staining Buffer Kit (Tonbo, US). Briefly, pelleted cells were resuspended within 1mL permeabilization solution and incubated 15min at RT in dark. Cells were then washed 2 times using Flow Cytometry Perm Buffer form the above-mentioned kit.

Following staining, cells were washed and resuspended in 1mL Maxpar Fix and Perm Buffer (Fluidigm, Inc Canada) with 1:4000 of Iridium intercalator (pentamethylcyclopentadienyl‐Ir (III)‐dipyridophenazine, [Fluidigm, Inc Canada]) for incubation overnight at 4°C. Cells were then frozen at -80°C until analysis.

### Mass cytometry acquisition

The day of analysis, cells were washed and resuspended in Maxpar Cell Acquisition Solution (Fluidigm, Inc Canada), a high-ionic-strength solution, mixed with 10% of EQ Beads (Fluidigm, Inc Canada) immediately before acquisition. Cell events were acquired on the HELIOS mass cytometer and CyTOF software version 6.7.1014 (Fluidigm, Inc Canada) at the Cytometry Platform of La Pitié-Salpétrière Hospital (CyPS). Mass cytometry standard files produced by the HELIOS system were normalized using the CyTOF Software v. 6.7.1014. This method normalizes the data to a global standard determined for each log of EQ beads.

### Mass cytometry data analysis

Mass cytometry standard files analysis was done using OMIQ data analysis software (www.omiq.ai).

Events are gated according to 191Ir positivity, corresponding to stained cells. This gate enables removing events that do not correspond to cells of the sample. All 140Ce-positive events, corresponding to calibration beads, are removed from the analysis. A gate is then placed on events negative for 195Pt which has been used for viability staining. Additional gates are established to remove contaminants from Barium (138Ba), Lanthanum (139La), Osmium (189Os),

Uniform manifold approximation and projection (UMAP) algorithm (83) was used with “2 dimensions” parameters.

### Adoptive transfer of ProTcell into newborn NSG mice

A total of 1x10^6^ ProTcells generated from CB HSPCs were intrahepatically transplanted into newborn NSG mice (between 1 and 4 days old). At 6-week-post-transplantation, the thymus was analyzed (using flow cytometry) for human cell engraftment and thymopoiesis. The study protocol was approved by the French Ministry of Higher Education and Research (reference: APAFIS 2010-2015090411495178v4, dated November 2nd, 2015 and APAFIS#29592–2020120216106476 v8, dated February 18th, 2021).

### Single cell RNA sequencing

Libraries from CB and mPB ProTcell samples were generated with Chromium Next GEM Single Cell 3’ GEM, Library & Gel Bead Kit (v3.1), following manufacturer’s instruction. Quality control was performed by High Sensitivity DNA assay, analyzed by Agilent 2100 Expert software.

Sequencing was made using Illumina technology (Illumina NextSeq 500) with libraries embedded in NovaSeq S2 FlowCell.

### scRNAseq: data processing

For read alignment and unique molecular identifiers (UMI) quantification, CellRanger software v6.1.2 (https://www.10xgenomics.com/support/software/cell-ranger/latest/release-notes/cr-release-notes) from the Chromium Single Cell Software Suite by 10x Genomics was used on Human cellranger reference 2020-A (genome GRCh38, annotations GENCODE v32/Ensembl 98).

Gene counts for each cell were quantified using the Cell Ranger ‘count’ command with default parameters.

The resultant gene expression matrix was imported into the R statistical environment (v4.2.2) for further analyses. Cell filtering, data normalization and clustering were carried out using the R package Seurat v4.3.0 (84). For each cell, the percentage of mitochondrial genes, number of total genes expressed, and cell cycle scores (S and G1 phases) were calculated. Cells with a ratio of mitochondrial versus endogenous gene expression >0.2 were excluded as putative dying cells. Cells expressing <500 or >8,000 total genes were also discarded as putative poorly informative cells and multiplets, respectively. Putative doublets were identified and discarded using scDblFinder R package (v1.4.0) (85) by imputing doublet rates (dbr) equal to 0.05. Dbr was established in agreement with the number of loaded cells and following the 10X Genomics guidelines. Cell cycle scores were calculated using the ‘CellCycleScoring’ function that assigns to each cell a score based on the expression of the S and G2/M phase markers and stores the S and G2/M scores in the metadata along with the predicted classification of the cell cycle state of each cell. Counts were normalized using Seurat function ‘NormalizeData’ with default parameters. Expression data were then scaled using the ‘ScaleData’ function, regressing on the number of unique molecular identifier, the percentage of mitochondrial gene expression and the difference between S and G2M scores. By using the most variable genes, dimensionality reduction was then performed with principal component analysis (PCA) by calculating 50 principal components (PCs) and selecting the top 30 PCs.

### scRNAseq: batch correction

PCA embeddings were corrected for sample batch by applying the Harmony algorithm (v0.1.0) (86), implemented by ‘RunHarmony’ function using the first 30 PCA dimensions and default theta (theta = 3). UMAP dimensionality reduction (87) was performed on the calculated PCs to obtain a two-dimensional representation for data visualization.

### scRNAseq: graph based clustering and differential expression

Cell clusters were identified using the Louvain algorithm at resolution r = 0.6, implemented by the ‘FindCluster’ function of Seurat. To find the differentially expressed genes from each cluster, the ‘FindAllMarkers’ function (iteratively comparing one cluster against all the others) from the Seurat package was used with the following parameters: adjusted P values <0.05, average log FC >0.25, and percentage of cells with expression >0.1. Dot plot was constructed using ‘DotPlot’ function in Seurat. Density plots were constructed using the ‘Plot_Density_Custom’ function from R packages scCustomize (v2.0.1) (88) and the Bioconductor/R Nebulosa package (89).

### scRNAseq: pseudotime analysis

Single cell pseudotime trajectories were computed with the R package Slingshot (v2.6.0) (90), which exploits previously computed cell clusters and focuses on the transcriptional lineage that describes the progressive transition from cluster 0 to cluster 6 by identifying genes whose expression changes along that trajectory. The differential expressed genes along the pseudotime tracjectory were analysed using the Bioconductor/R package tradeseq (v1.12) (91).

### Statistical analysis

Statistical analyses of results were conducted using GraphPad Prism (Between v9.4.0.673 and 10.4.0).

## Data Availability

The datasets presented in this study can be found in online repositories. The names of the repository/repositories and accession number(s) can be found below: https://www.ncbi.nlm.nih.gov/, GSE292844.

## Supplementary material

Supplementary Table 1 ProTcells were analyzed by scRNAseq and gene expression was assessed in CD7^+^ clusters. Differentially expressed genes were listed for each cluster, as compared to the other clusters.

Supplementary Figure 1
**(A)** UMAP projection showing each individual integrated CB and mPB samples from the scRNAseq analysis. **(B)** UMAP projection depicting the different clusters identified in the whole dataset from the scRNAseq analysis (resolution 0.6). **(C)** Contour plot of flow cytometry analysis depicting the expression of CD7 and CD34 from representative CB and mPB CD34^+^ cells used for ProTcell production. **(D)** Feature plot showing the CD7 expression distribution in the whole dataset analyzed by scRNAseq. **(E)** Contour plot of flow cytometry analysis depicting the expression of CD7 and CD3 from a representative CB ProTcell. **(F)** Feature plots illustrating the expression of RAG1 and RAG2 genes across the CD7^+^ ProTcells analyzed by scRNAseq.

Supplementary Figure 2 Heatmap comparing the expression levels of the top 10 differentially expressed genes between CD7^+^ ProTcell clusters from the scRNAseq analysis. The color scale indicates the average expression levels of these genes in each cluster (purple represents low; yellow represents high).

Supplementary Figure 3 ProTcells were analyzed by scRNAseq and gene expression was assessed in CD7^+^ clusters. **(A)** Density plots illustrating the gene expression of developing thymocytes markers CD5, CD1E and PTCRA. **(B)** Violin plots showing the normalized gene expression level and **(C)** density plots illustrating the gene expression of thymus homing molecules CXCR4, SELL and ITGA4. **(D)** Violin plots showing the normalized gene expression level of several lymphoid markers. Density plots illustrating the gene expression of **(E)** ILC progenitor associated markers IL1R, ITGB7, PTGDR2 and CCR6 and **(F)** LEF1, a T cell-related marker.

Supplementary Figure 4
**(A)** ProTcells were analyzed by scRNAseq and progenitor differentiation trajectories were calculated by Slingshot across CD7^+^ clusters (**Figure 2F**). Feature plots illustrating the gene expression of the top markers differentiating between lineage 2 (toward T cell-primed cells) and lineage 1 (ILC-oriented cells). **(B)** Overlay histogram of CyTOF analysis showing the level of expression of BCL11B in CD7^+^ CD161^-^, CD7^+^ CD161^+^ and CD7^+^ CD161^+^ CD56^+^ CB ProTcell subsets.

Supplementary Figure 5 CB ProTcells were FACS-sorted based on their CD161 expression for further *in vitro* T and NK cell differentiation assays and *in vivo* transplantation experiments. **(A)** Representative contour plots of flow cytometry analysis depicting the CD3 and CD56 expression in CD161^+^ or CD161^-^ ProTcells after 2 weeks of NK differentiation. **(B)** Representative dot plots of flow cytometry analysis depicting the expression of CD4 and CD8 (left), CD3 and TCRαβ (middle) and CD3 and TCRγδ (Right) in differentiating progenitors over the 4-week-co-culture of CD161^+^ and CD161^-^ ProTcells with OP9-DLL1 for T cell differentiation. The black frames delineate the DP population. Line plots depicting the mean ± SD of the **(C)** proportion and **(D)** number of CD4^+^ CD8^+^ developing T cell from CD161^+^ and CD161^-^ ProTcells throughout the 4-week-co-culture with OP9-DLL1. **(E)** Representative contour plots of flow cytometry analysis depicting the T cell differentiation within the thymus witnessed by the co-expression of CD3 and TCRαβ (left) and the single expression of CD4 or CD8 (right), 6 weeks after CD161^+^ and CD161^-^ ProTcells transplantation in NSG neonates (< 4-day-old).

Supplementary Figure 6
**(A)** Illustration of the EGFP-BAC used to generate the BCL11B reporter hPSC line. EGFP-BAC reporter is integrated at exon 1 of endogenous *BCL11B* gene, thus resulting in a monoallelic disruption of this gene. **(B)** Representative contour plot of flow cytometry analysis illustrating *BCL11B*-EGFP and CD11b expression in *BCL11B*-EGFP^neg^-sorted progenitors after 5 days of co-cultured with OP9 for myeloid differentiation. **(C)** Bar plot representing the mean ± SD of *BCL11B*-EGFP expression level within CD11b cells derived from *BCL11B*-EGFP^neg^ progenitors co-culture 5 days with OP9 for myeloid differentiation. **(D)** Representative contour plots of flow cytometry analysis depicting *BCL11B*-EGFP expression in differentiating cells derived from *BCL11B*-EGFP^neg^ and *BCL11B*-EGFP^+^-sorted progenitors after 7 days of co-culture with OP9-DLL4 for T cell differentiation.
