# Supplementary material for: Ex vivo-generated lymphoid progenitors encompass both T cell and innate lymphoid cell fates
Source: Front Immunol. 2025 Jul 23;16:1617707. doi: 10.3389/fimmu.2025.1617707 (PMC12325321; doi:10.3389/fimmu.2025.1617707)
Supplement: Supplementary Figure 1 — (A) UMAP projection showing each individual integrated CB and mPB samples from the scRNAseq analysis. (B) UMAP projection depicting the different clusters identified in the whole dataset from the scRNAseq analysis (resolution 0.6). (C) Contour plot of flow cytometry analysis depicting the expression of CD7 and CD34 from representative CB and mPB CD34+ cells used for ProTcell production. (D) Feature plot showing the CD7 expression distribution in the whole dataset analyzed by scRNAseq. (E) Contour plot of flow cytometry analysis depicting the expression of CD7 and CD3 from a representative CB ProTcell. (F) Feature plots illustrating the expression of RAG1 and RAG2 genes across the CD7+ ProTcells analyzed by scRNAseq. [file Presentation1.pptx]

## Slide 1
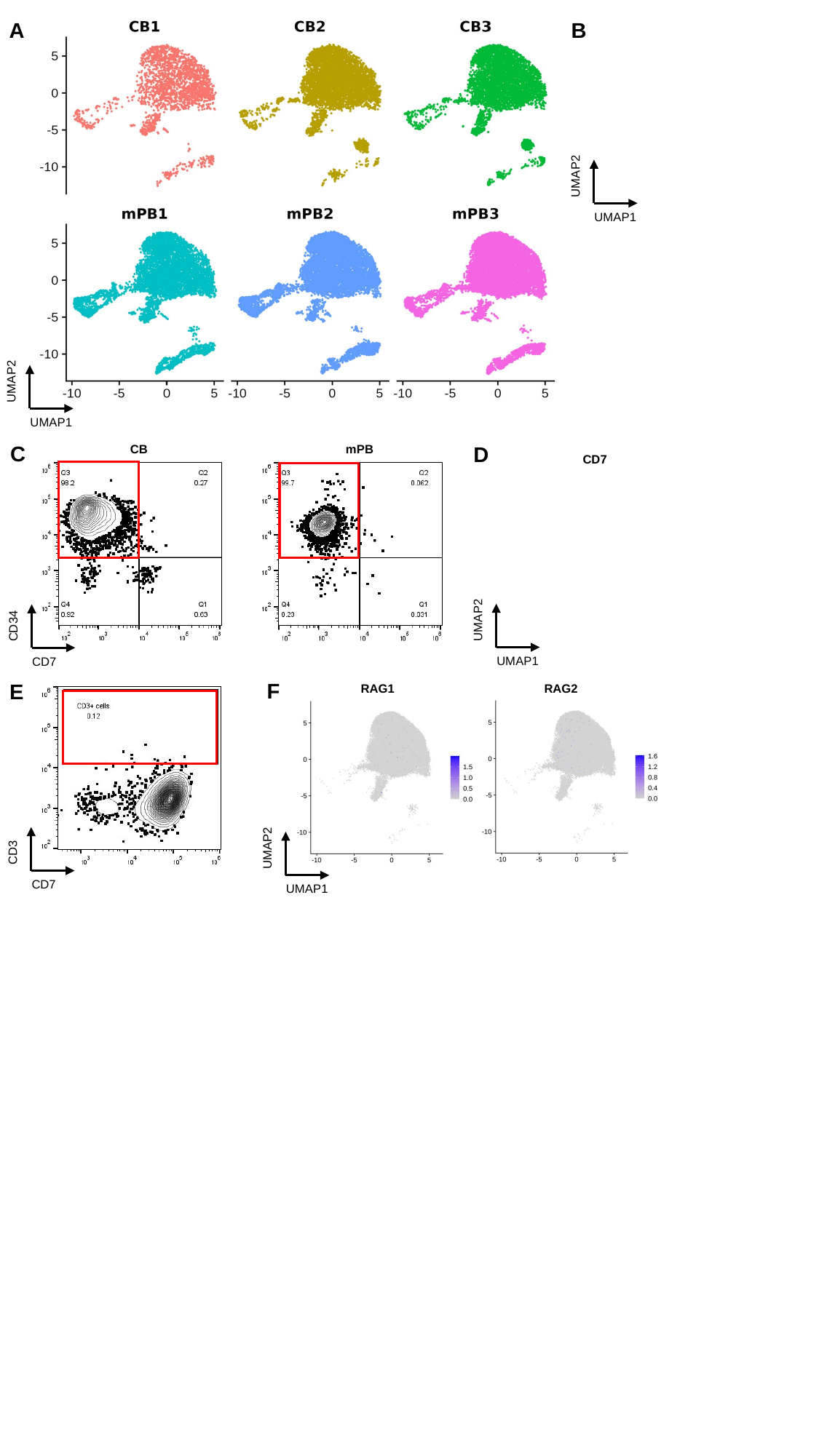

B
A
UMAP2
UMAP1
UMAP2
UMAP1
C
D
CB
mPB
CD7
UMAP2
UMAP1
CD34
CD7
F
RAG1
RAG2
UMAP2
UMAP1
E
CD3
CD7

## Slide 2
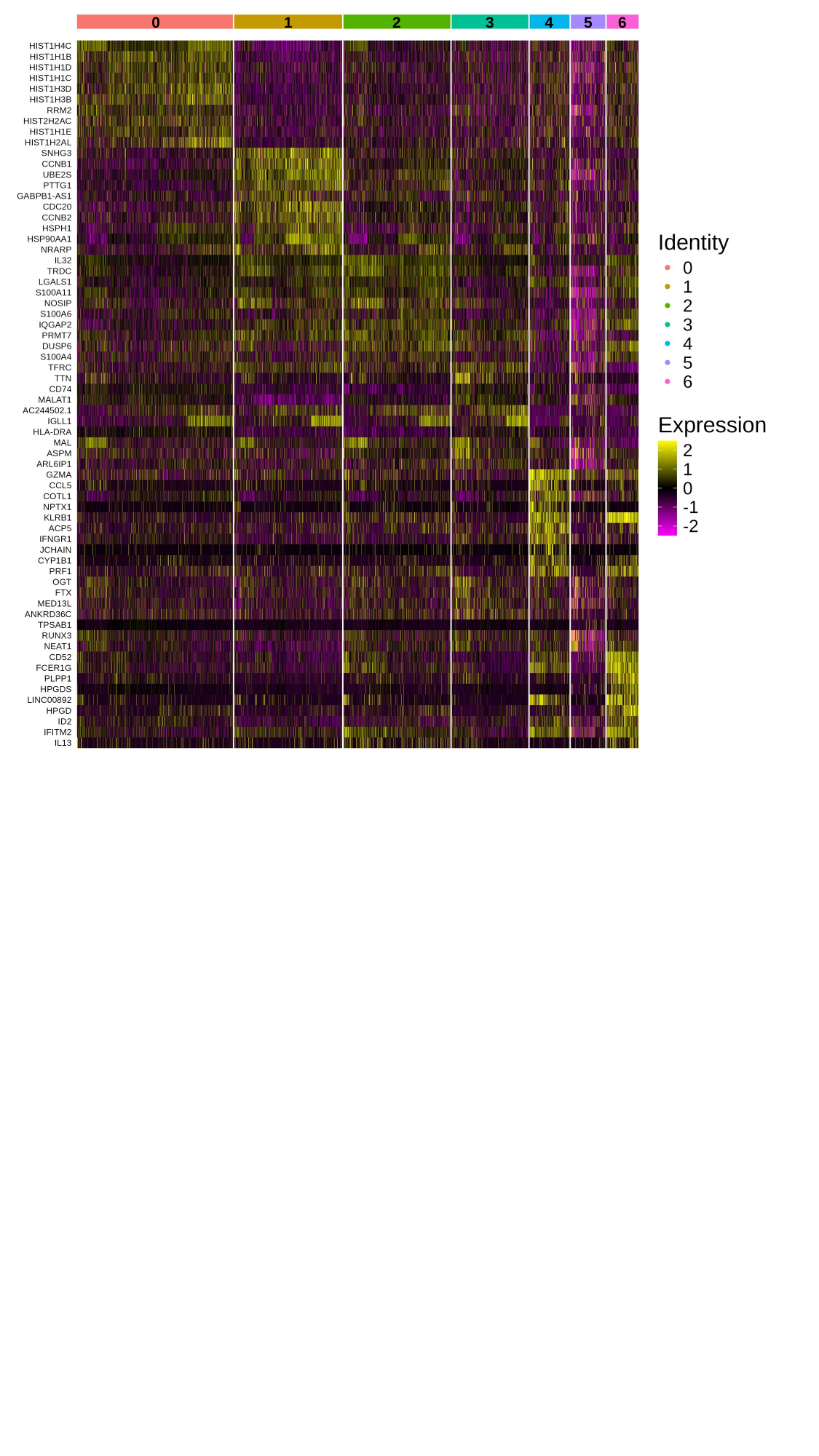

0
1
2
3
4
5
6

## Slide 3
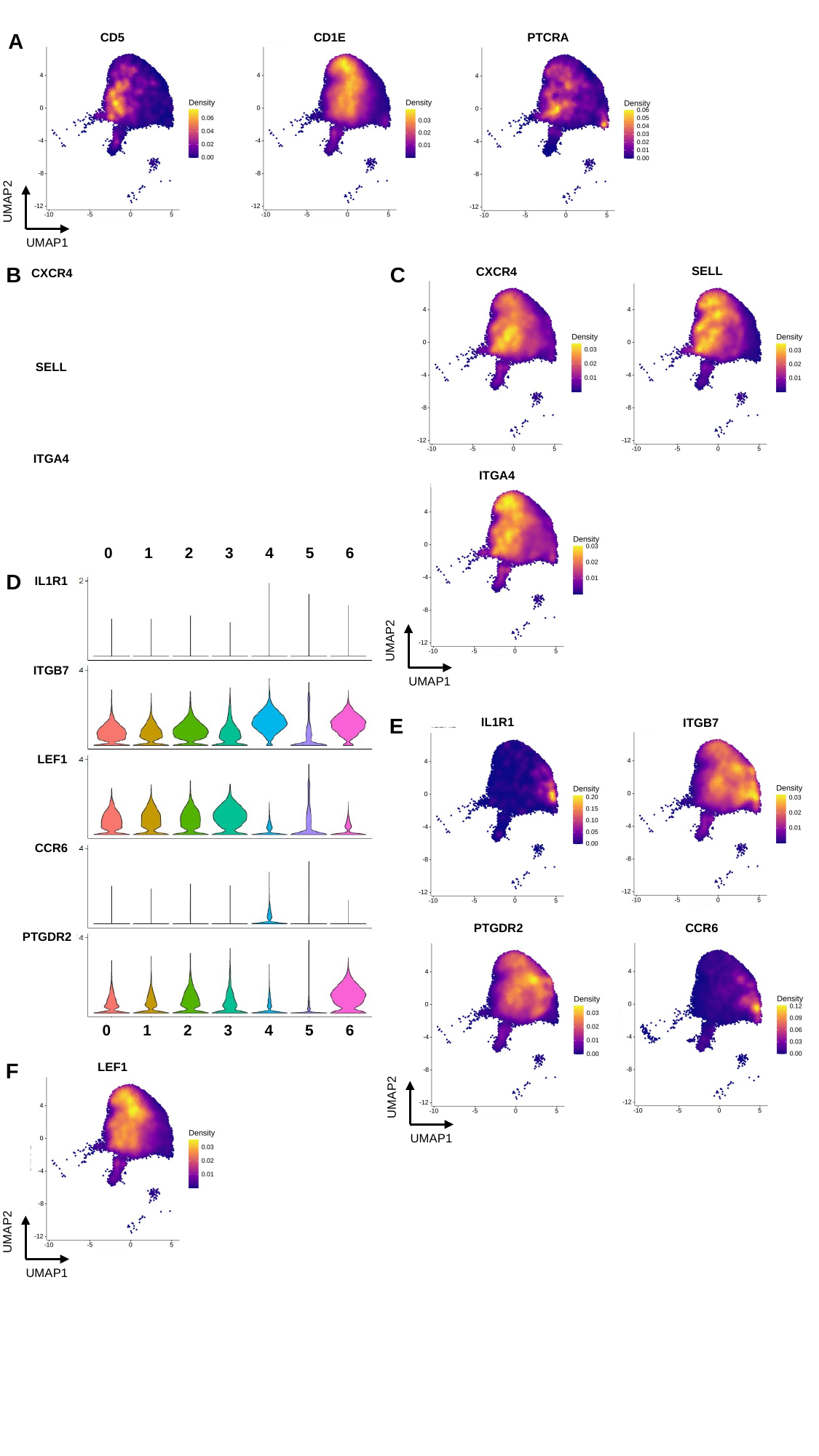

A
PTCRA
CD5
CD1E
UMAP2
UMAP1
B
C
SELL
CXCR4
CXCR4
SELL
ITGA4
ITGA4
| 0 | 1 | 2 | 3 | 4 | 5 | 6 |
| --- | --- | --- | --- | --- | --- | --- |
D
IL1R1
UMAP2
UMAP1
ITGB7
E
IL1R1
ITGB7
LEF1
CCR6
PTGDR2
CCR6
PTGDR2
| 0 | 1 | 2 | 3 | 4 | 5 | 6 |
| --- | --- | --- | --- | --- | --- | --- |
F
LEF1
UMAP2
UMAP1
UMAP2
UMAP1

## Slide 4
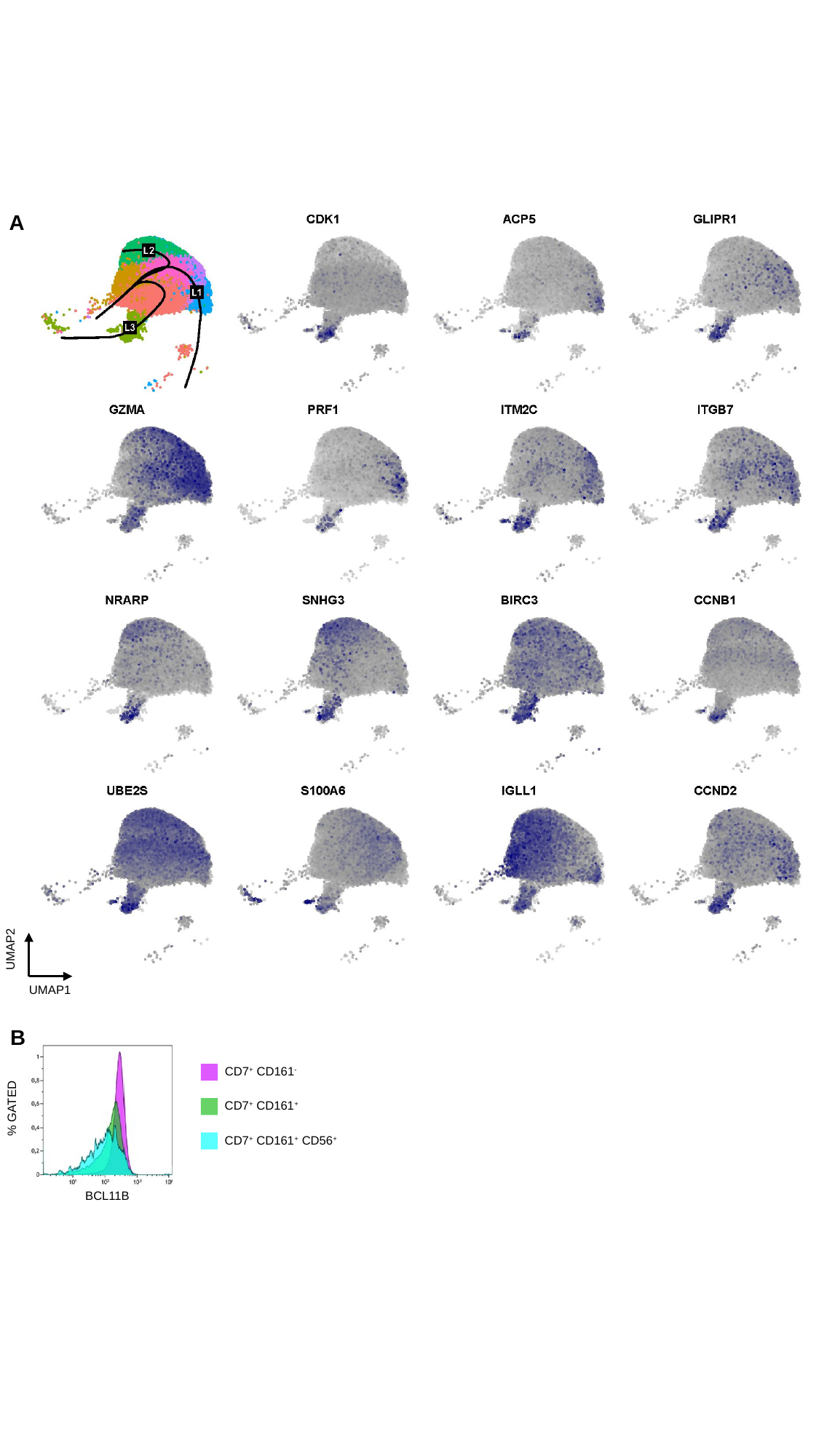

A
UMAP2
UMAP1
B
CD7+ CD161-
CD7+ CD161+
CD7+ CD161+ CD56+
% GATED
BCL11B

## Slide 5
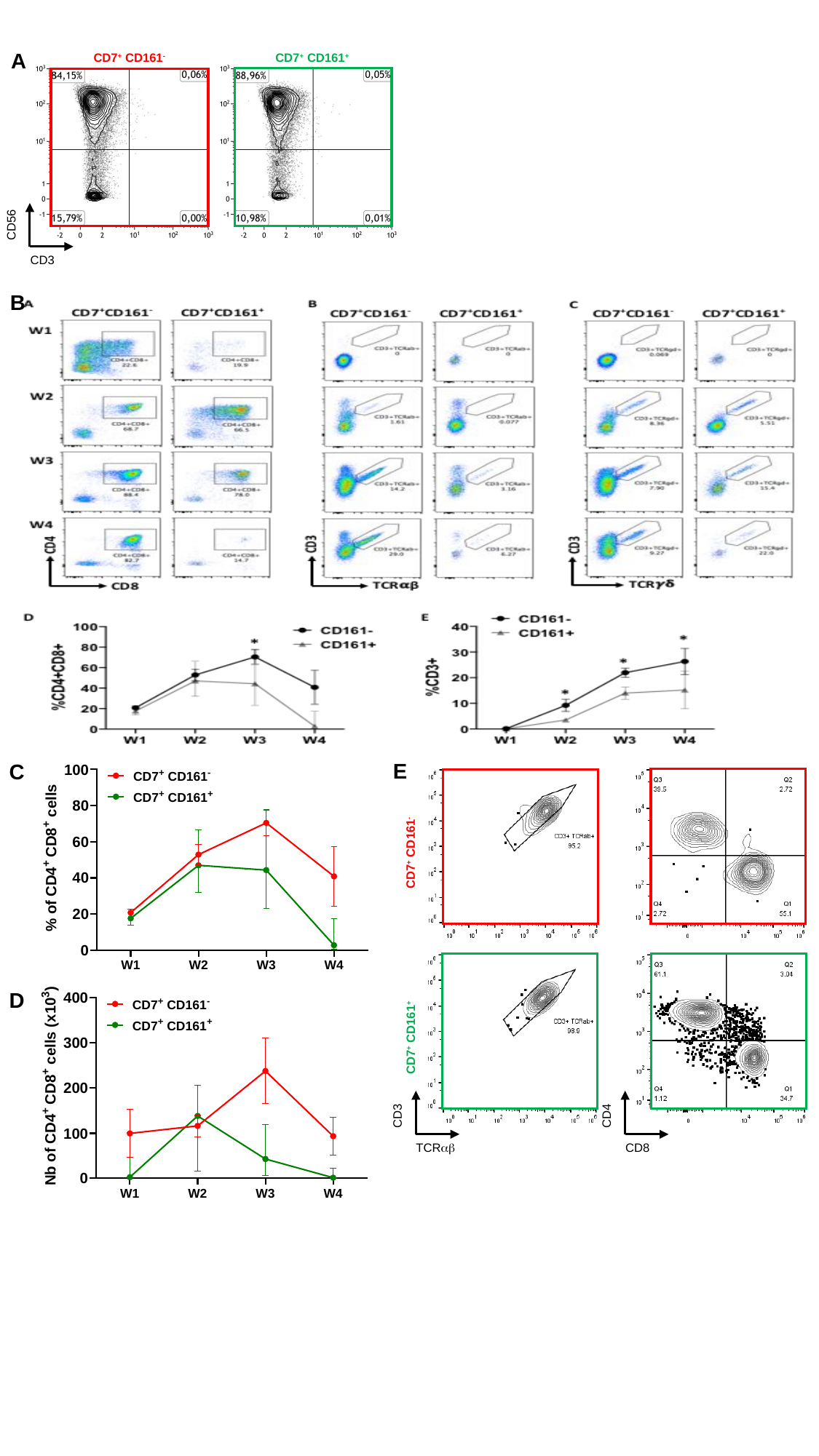

A
CD7+ CD161-
CD7+ CD161+
CD56
CD3
B
E
C
CD7+ CD161-
D
CD7+ CD161+
CD3
TCRab
CD4
CD8

## Slide 6
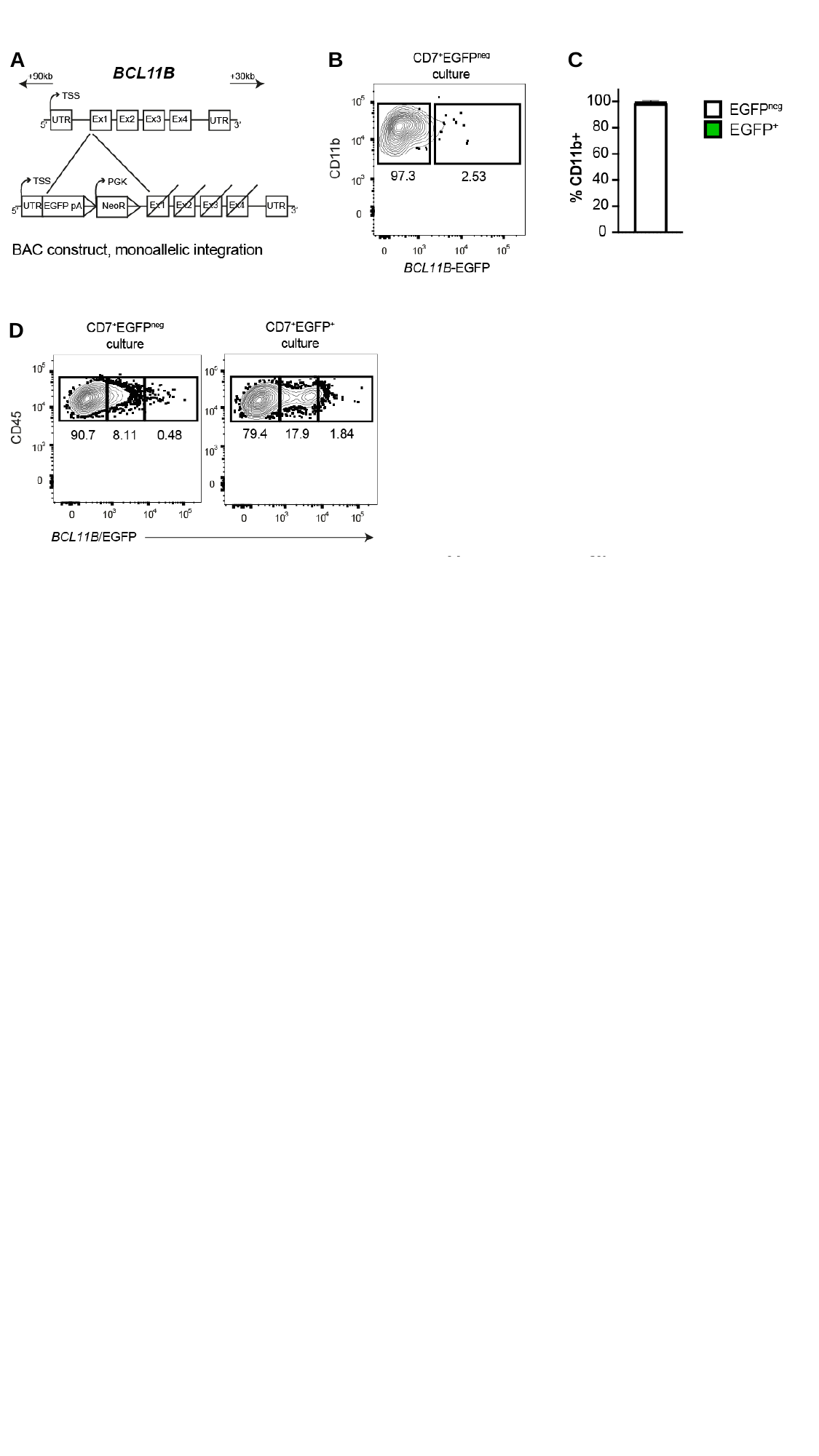

A
B
C
D
